# Oxidized linoleic acid metabolites regulate neuronal morphogenesis *in vitro*

**DOI:** 10.1016/j.neuint.2023.105506

**Published:** 2023-02-08

**Authors:** Felipe da Costa Souza, Ana Cristina G. Grodzki, Rhianna K. Morgan, Zhichao Zhang, Ameer Y. Taha, Pamela J. Lein

**Affiliations:** a Department of Food Science and Technology, College of Agriculture and Environmental Sciences, University of California, Davis, CA, USA; b Department of Molecular Biosciences, School of Veterinary Medicine, University of California, Davis, CA, USA

**Keywords:** OXLAMs, Neuronal morphogenesis, Primary neuronal cultures, Linoleic acid, Oxylipins, Dendritic arborization

## Abstract

Linoleic acid (LA, 18:2n-6) is an essential nutrient for optimal infant growth and brain development. The effects of LA in the brain are thought to be mediated by oxygenated metabolites of LA known as oxidized LA metabolites (OXLAMs), but evidence is lacking to directly support this hypothesis. This study investigated whether OXLAMs modulate key neurodevelopmental processes including axon outgrowth, dendritic arborization, cell viability and synaptic connectivity. Primary cortical neuron-glia co-cultures from postnatal day 0–1 male and female rats were exposed for 48h to the following OXLAMs: 1) 13-hydroxyoctadecadienoic acid (13-HODE); 2) 9-hydroxyoctadecadienoic acid (9-HODE); 3) 9,10-dihydroxyoctadecenoic acid (9,10-DiHOME); 4) 12(13)-epoxyoctadecenoic acid (12(13)-EpOME); 5) 9,10,13-trihydroxyoctadecenoic acid (9,10,13-TriHOME); 6) 9-oxo-octadecadienoic acid (9-OxoODE); and 7) 12,13-dihydroxyoctadecenoic acid (12,13-DiHOME). Axonal outgrowth, evaluated by Tau-1 immunostaining, was increased by 9-HODE, but decreased by 12,13-DiHOME in male but not female neurons. Dendrite arborization, evaluated by MAP2B-eGFP expression, was affected by 9-HODE, 9-OxoODE, and 12(13)-EpOME in male neurons and, by 12(13)-EpOME in female neurons. Neither cell viability nor synaptic connectivity were significantly altered by OXLAMs. Overall, this study shows select OXLAMs modulate neuron morphology in a sex-dependent manner, with male neurons being more susceptible.

## 1. Introduction

Linoleic acid (LA, 18:2n-6) is an essential polyunsaturated fatty acid (PUFA) that is required at a minimum 1–2% of daily calories to support optimal infant development (Hansen et al., 1958). LA levels in the food supply have increased from 2 to 7% of daily calories over the past century (Blasbalg et al., 2011), resulting in an increase in human breast milk LA composition and exposure to infants. Milk LA composition has increased from 7% to 12% of total fatty acid during the 70s and 80s (Gibson and Kneebone, 1981; R. G. Jensen et al., 1992; Putnam et al., 1982), with most recent findings reporting up to 20% LA of total fatty acids (Mendonca et al., 2017). Similarly, LA composition in infant formulas can vary from 9 to 20% of total fatty acids (Mendonca et al., 2017).

Observational studies in humans have reported an inverse association between breast milk LA levels and both cognitive scores and verbal IQ, suggesting that exposure to excess LA in early life might impair normal brain development (Bernard et al., 2015). In another study using magnetic resonance imaging (MRI) of 1553 mother and child pairs, higher levels of dietary LA during pregnancy were associated with reduced infant white matter volume at 9 to 11 years of age (Zou et al., 2021).

The effects of LA in the brain are thought to be mediated by enzymatically=generated oxidized LA metabolites known as ‘OXLAMs’. LA itself is not abundant in the rat and human brain (<2% of total fatty acids) (DeMar et al., 2006; Lien et al., 1994; Martínez and Mougan, 2002; Sanders et al., 1984), because it is beta-oxidized, recycled into saturated fatty acids and cholesterol, or converted into OXLAMs upon entering the brain (Cunnane et al., 1994; Green and Yavin, 1993; Hassam et al., 1975; Taha et al., 2018). Enzymes involved in OXLAM synthesis from LA include lipoxygenase (LOX), cytochrome P450 (CYP450) and soluble epoxide hydrolase (sEH) enzymes (Earles et al., 1991; Funk and Powell, 1983; Laneuville et al., 1995; Reinaud et al., 1989).

OXLAMs may play a critical role in early brain development. Recently, we reported that OXLAMs constitute 47–52% of oxidized fatty acids (i.e., oxylipins) in whole brain of 0–1-day old male and female rat pups (Hennebelle et al., 2020). This is at least 10 times higher than reported in adult rat brain (5–7% OXLAMs), suggesting a potentially important need for OXLAMs in the developing rat brain (Hennebelle et al., 2019, 2020; Taha et al., 2018). Additionally, in a prior study, we found that 13-hydroxyoctadecadienoic acid (13-HODE), the most abundant OXLAM found in the developing brain, significantly increased axonal outgrowth in male cortical neuron-glia co-cultures at a physio-logically relevant concentration of 100 nM (Hennebelle et al., 2019).

**Fig. 1.**
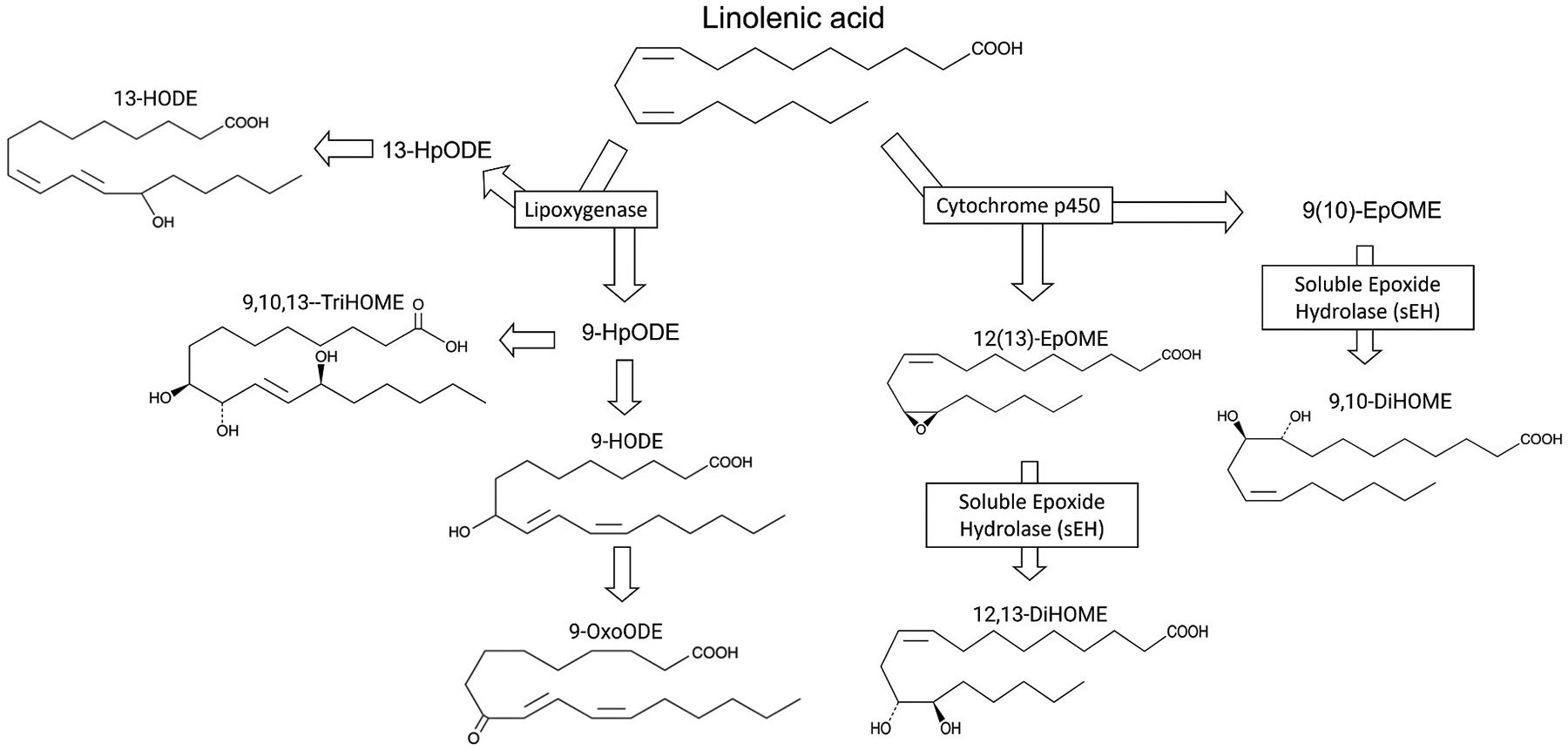
Overview of OXLAM structures tested in this study. Main enzymatic pathways that produce OXLAMs are illustrated. Structures of the OXLAMs tested in this study, specifically, 13-hydroxyoctadecadienoic acid (13-HODE), 9-hydroxyoctadecadienoic acid (9-HODE); 9,10-dihydroxyoctadecenoic acid (9,10-DiHOME); 12(13)epoxyoctadecenoic acid (12(13)-EpOME); 9,10,13-trihydroxyoctadecenoic acid (9,10,13-TriHOME); 9-oxo-octadecadienoic acid (9-OxoODE); and 12,13-dihydroxyoctadecenoic acid (12,13-DiHOME) are shown. Structures of intermediary metabolites and their enzymes were intentionally omitted. The structures were adapted from the supplier’s datasheet of all the standards used in this study.

**Fig. 2.**
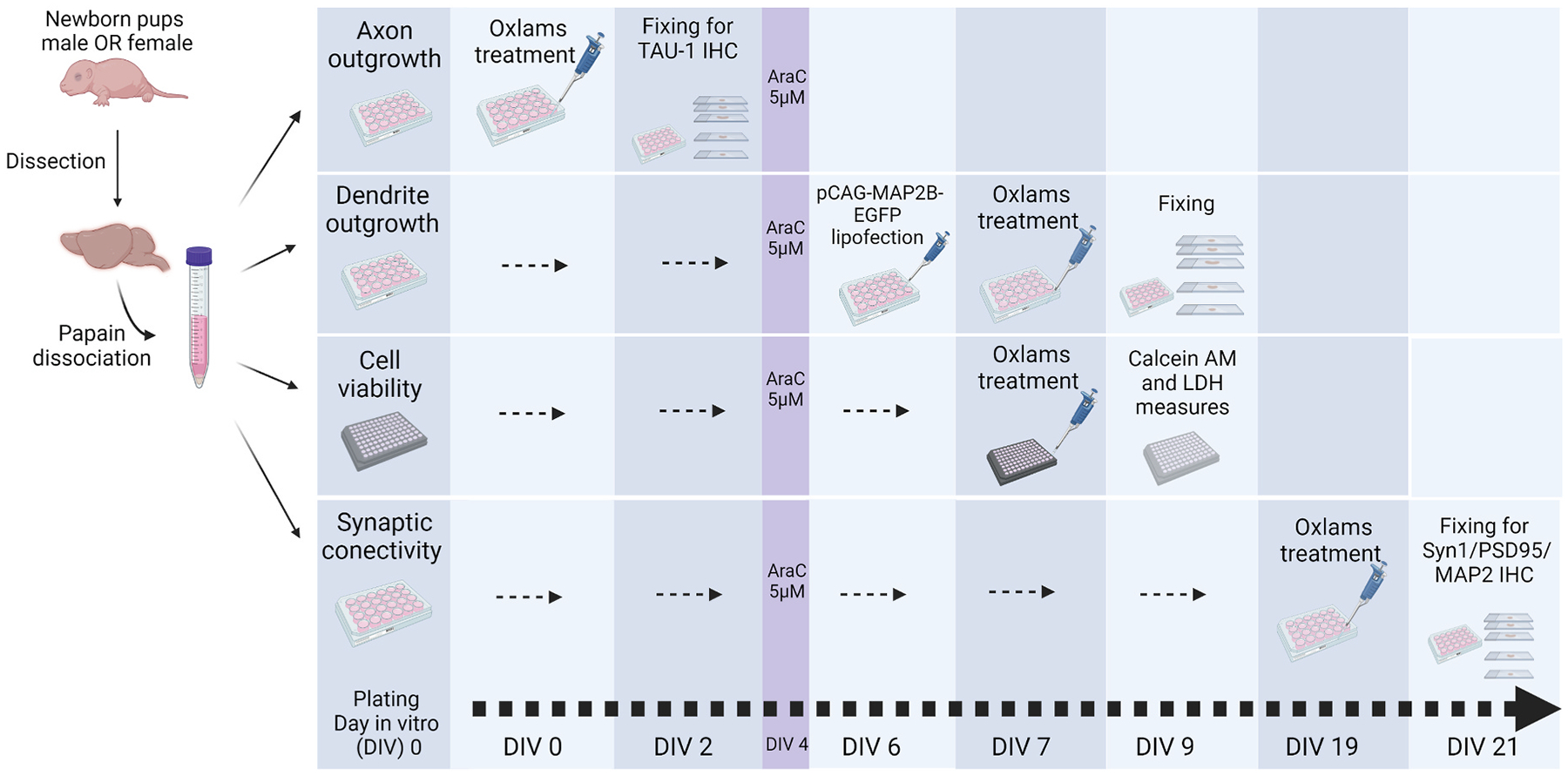
Flowchart of experimental design for primary rat cortical neuron-glia co-cultures. Postnatal day 0 or 1 pups neocortices were dissected, male and female tissues separately pooled, and cells enzymatically dissociated and plated at assay-specific densities. Flowchart for each assay designates days *in vitro* (DIV) of treatment and collection for endpoint assessment. Four to eight brains were pooled for male vs. female cultures from each of three independent dissections. Synaptic connectivity was examined in cultures from four independent dissections.

Here, we expand on our prior study by investigating the effect on axonal growth and dendritic arborization of 13-HODE as well as six other OXLAMs (Fig. 1): 9-hydroxyoctadecadienoic acid (9-HODE); 9,10-dihydroxyoctadecenoic acid (9,10-DiHOME); 12(13)-epoxyoctadecenoic acid (12(13)-EpOME); 9,10,13-trihydroxyoctadecenoic acid (9,10,13-TriHOME); 9-oxo-octadecadienoic acid (9-OxoODE); and 12,13-dihydroxyoctadecenoic acid (12,13-DiHOME). Each of the OXLAMs tested here was previously detected in the brain of male and female rat pups (Hennebelle et al., 2020). We also explored the effects of these compounds on synaptic connectivity and cell viability. Axon outgrowth, dendritic arborization, cell viability and synaptic connectivity were analyzed in sex segregated primary cortical neuron-glia co-cultures derived from 0–1-day old male and female rat pups (Fig. 2).

## 2. Methods

### 2.1. Animals

Animal procedures were approved by the University of California, Davis Institutional Animal Care and Use Committee (IACUC Protocol Number 22319). Timed pregnant Sprague-Dawley rats (RRID: MGI_5651135) were purchased from Charles River Laboratory (Hollister, CA, USA). The rats arrived at the vivarium at least 16 d post-conception (E16) and were individually housed with corncob bedding. The room was kept at a constant temperature (~22 °C) with a 12-h light-dark cycle and food and water provided *ad libitum*. Dams received a 2018 Teklad global 18% protein diet (Envigo RMS. Inc, IN, USA, Cat#2018) upon arrival. The diet contained 186 g/kg protein, 62 g/kg fat, 589 g/kg carbohydrates, and 53 g/kg ash. The fatty acid composition of the diet, as previously measured by gas-chromatography (Hennebelle et al., 2020), was 13.3% palmitic acid (16:0), 3.1% stearic acid (18:0), 20.4% oleic acid (18:1n-9), 55.6% LA and 6.7% alpha-linolenic acid. These values were consistent with the manufacturer’s report.

### 2.2. Primary cortical neuron-glia co-cultures

Male and female-specific primary cortical neuron-glia co-cultures were prepared from male and female pups on postnatal day (PND) 0 or PND 1 with pup sex determined by measuring the anogenital distance as previously described (Hennebelle et al., 2020; Sethi et al., 2017). At least three independent dissections were conducted for each assay, and each independent dissection consisted of 4–6 male and female pups from the same mother. No exclusion criteria were pre-determined, and no animals were excluded. The pups were anesthetized with cold and then euthanized by decapitation and their brains rapidly excised. Neocortices from male and female pups were independently pooled into male and female pools. Neocortical tissues were dissected in ice-cold Hanks’ Balanced Salt Solution (Gibco, Thermo Fisher Scientific, Waltham, MA, USA; Cat #14185–052) supplemented with 1 M HEPES buffer (pH 7.55; Sigma-Aldrich, St. Louis, MO, USA; Cat #BP310–500) and then incubated at 37 °C for 23 min in Hibernate A (Gibco, Thermo Fisher Scientific; Cat #A1247501) containing 2.3 mg/mL papain (Worthington, Lakewood, NJ, USA; Cat #LS003119) and 95 μg/mL DNase (Sigma-Aldrich; Cat #D5025). The papain/DNase solution was removed, and the cortical tissue rinsed with Neurobasal Plus medium (Thermo Fisher Scientific; Cat #A3582901) supplemented with 2% B27 (Thermo Fisher Scientific; Cat #A3582801), 1% GlutaMAX (Thermo Fisher Scientific; Cat #35050–061), 10% horse serum (Gibco, Thermo Fisher Scientific; Cat #26050–088) and 1 M HEPES buffer. The tissue was then physically triturated using bent tips pipettes (Bellco, Vineland, NJ, USA; Cat #:1273–40004). Cells were counted using a Cellometer Auto T4 Automated Cell Counter (Nexcelom Bioscience LLC, MA, USA) and plated on sterile glass coverslips (BellCo; Cat #1943–10012A) precoated with 500 μg/mL poly-L-lysine (Sigma-Aldrich; Cat #P1399). Cells were seeded at different cell densities for different assays and allowed to settle and attach at 37 °C under 5% CO_2_. Three to 4 h post-plating, culture medium was replaced with Neurobasal Plus basal medium supplemented with 2% B27 and 1% GlutaMAX. On day *in vitro* (DIV) 4, half of the conditioned medium was replaced by fresh Neurobasal Plus basal medium supplemented with 2% B27 and 1% GlutaMAX and 10 μM cytosine-arabinoside (AraC) at a final concentration of 5 μM AraC to inhibit glial proliferation (Lesslich et al., 2022).

### 2.3. Treatments

13-HODE (Cayman Chemical, Ann Arbor, MI, USA; Cat #38600); 9-HODE (Cayman Chemical; Cat #38400); 9,10-DiHOME (Cayman Chemical; Cat #53400); 12(13)-EpOME (Cayman Chemical; Cat #52450); 9,10,13-TriHOME (Cayman Chemical; Cat # 26768); 9-OxoODE (Cayman Chemical; Cat #38420); and 12,13-DiHOME (Cayman Chemical; Cat #10009832) were dissolved as 1 mM stocks in absolute ethanol. Ultra-high pressure liquid chromatography coupled to tandem mass spectrometry (UPLC-MS/MS) was used to confirm the purity of all stock solutions (Supplemental file 1, Fig S1) as previously described (Hennebelle et al., 2020). From these stocks, each compound was diluted in Neurobasal Plus basal medium supplemented with 2% B27 and 1% GlutaMAX to a final concentration of 1, 10, 50, 100, 500, or 1000 nM. The final ethanol concentration was 0.1%. Media containing each of the OXLAMs was directly added to neuron-glia co-cultures.

Vehicle controls were treated with 0.1% ethanol in Neurobasal Plus basal medium supplemented with 2% B27 and 1% GlutaMAX. Brefeldin-A (5 μM), which was used as a positive technical control for the neurite retraction assay (Jareb and Banker, 1997), was also diluted in Neurobasal Plus basal medium supplemented with 2% B27 and 1% GlutaMAX. Cultures were exposed to Brefeldin-A and ethanol 0.1% as described above for OXLAMs.

For dendrite growth and cell viability assays, OXLAMs were diluted in medium at twice the final concentration; one volume of this medium with 2X OXLAMs was added to each well containing the same volume of conditioned medium to yield a final OXLAM concentration of 1, 10, 50, 100, 500, or 1000 nM.

### 2.4. Quantification of axonal growth

Dissociated cortical cells were seeded at a concentration of 26,500 cells/cm^2^ on 24-well plates with glass coverslips pre-coated with poly-L-lysine, with three wells per treatment. Plating medium was replaced with Neurobasal Plus basal medium containing 2% B27, 1% GlutaMAX, and the appropriate concentration of each OXLAM (1–1000 nM) or controls (vehicle or Brefeldin-A) during the 3–4 h post-plating media change on DIV 0. The total incubation period with or without OXLAMs was 48 h. B27 media contains bovine serum albumin, which has been shown to bind free oxylipins and minimize their degradation in cell culture for at least 48 h (Maddipati and Zhou, 2011). After 48 h of OXLAM exposure, the cultures were fixed with 4% paraformaldehyde (Sigma-Aldrich; Cat #441244) in phosphate buffer (PB, 3.6 mM Na_2_HPO_4_, 1.4 mM NaH_2_PO_4_, pH 8) for 45 min, rinsed three times with phosphate-buffered saline (PBS, 3.6 mM Na_2_HPO_4_, 1.4 mM NaH_2_PO_4_, 150 mM NaCl; pH 7.2) for 5 min and permeabilized with 0.2% Triton X-100 (Sigma-Aldrich; Cat #T9284) in PBS for 5 min. Permeabilized cells were blocked in 5% bovine serum albumin (BSA, Sigma-Aldrich; Cat #A9647) in PBS for 1 h. To selectively label axons, cells were then incubated overnight at 4 °C with the primary antibody anti-Tau1 (Millipore, Billerica, MA, USA; Cat #MAB3420; RRID: AB_2139842) diluted 1:1000 in 5% BSA in PBS. Cultures were washed three times with PBS and incubated for 1 h at room temperature with secondary antibody, fluorescein goat anti-mouse IgG (Invitrogen, Thermo Fisher Scientific; Cat #A21131; RRID: AB_2535771) diluted 1:1000 in 5% BSA in PBS. Slides were mounted in Invitrogen Prolong Gold Antifade Reagent with DAPI (Invitrogen, Thermo Fisher Scientific; Cat #P36935). Images of immunostained neurons were captured from at least twenty-five fields per well at 10x magnification, with three wells from three separate dissections per treatment per sex, using an automated high content imaging system (ImageXpress; Molecular Devices, Palo Alto, CA, USA). Automated image analysis of axonal outgrowth was performed using a cell scoring custom built journal on MetaXpress software (Molecular Devices; version 5.3.0.5) (Supplemental file 2, Fig S11).

### 2.5. Quantification of dendrite growth

Dissociated cortical cells were seeded at a concentration of 79,000 cells/cm^2^ on 24-well plates with glass coverslips pre-coated with poly-L-lysine, with three wells per treatment. The effect of OXLAMs on dendritic morphology was analyzed using Sholl analysis. Maximal expansion of the dendritic arborization in these cultures occurs between DIV 5 and 9 (Wayman et al., 2006). Therefore, on DIV 6, cultures were transfected with a plasmid encoding microtubule-associated protein 2B fused to enhanced green fluorescent protein (pCAG-MAP2B-EGFP, generously provided by Dr. Gary Wayman, University of Washington, Pullman, WA, USA) (Wayman et al., 2006). Cultures were lipofected for 2 h with 0.8 μG of plasmid using Lipofectamine-2000 Transfection Reagent (Invitrogen, Thermo Fisher Scientific; Cat # 11668030) following the manufacturer’s instructions. This protocol results in a low transfection efficiency (1–10% of neurons in the culture), which allows visualization of the entire dendritic arbor of individual neurons in a high density culture. On DIV7, cells were treated with OXLAMs or controls for 48 h. At DIV 9, cultures were fixed with 4% paraformaldehyde in PB for 45 min, rinsed three times with PBS for 5 min and mounted to glass slides using Prolong Gold Antifade Reagent with DAPI. Images of MAP2B-EGFP-labeled neurons were captured from at least twenty-five fields per well at 10x magnification, with three wells from three separate dissections per treatment per sex, using an automated high content imaging system (ImageXpress). The dendritic complexity of each individual neuron was quantified by Sholl analysis for each well using the automated unbiased extension of Omnisphero software as described by Schmuck et al. (2020). (Schmuck et al., 2020).

### 2.6. Cell viability

Primary cortical neuron-glia co-cultures were prepared as described above. Neocortical cells were plated at 79,000 cells/cm^2^ in flat-bottomed 96-well plates (Corning Inc; Cat #3606) pre-coated with poly-L-lysine. On DIV 7, cultures were treated with Neurobasal Plus basal medium supplemented with 2% B27, 1% GlutaMAX and either an OXLAM (1–1000 nM) or vehicle (0.1% ethanol). On DIV 9, cell viability was quantified by measuring lactate dehydrogenase (LDH) in the culture media using the CytoTox-ONE Homogenous Membrane Integrity Assay (Promega, Madison, WI, USA) following the manufacturer’s instructions. Cells treated with 0.2% Triton X-100 served as a technical control for reduced viability. The DIV 9 cells were co-stained with calcein-AM (5 μM, ThermoFisher Scientific) and Hoechst-33342 (1 μg/ml, Sigma-Aldrich) to identify live versus dead cells, respectively. Plates were imaged and the percent live cells quantified using the Image Express Micro XL high content imaging system (Molecular Devices; version 5.3.0.5) using a custom built journal in MetaXpress Software (Supplemental file 2, Fig S12). At least four fields were imaged per well at 10x magnification from five wells from three separate dissections per treatment per sex.

### 2.7. Quantification of synaptic connectivity

Primary cortical neuron-glia co-cultures were seeded at 52,000 cells/cm^2^ on glass coverslips pre-coated with poly-L-lysine in 24-well plates. The culture was maintained until DIV 18 with half of the medium exchanged for fresh medium every 4 d. On DIV 19, cultures were treated with Neurobasal Plus basal medium supplemented with 2% B27 and 1% GlutaMAX supplemented with the relevant concentration of each treatment (13-HODE; 9-HODE; 9,10-DiHOME; 12(13)-EpOME; 9,10,13-TriHOME; 9-OxoODE; 12,13-DiHOME) or vehicle (0.1% ethanol). After 48 h of exposure, cells were fixed with 4% paraformaldehyde in PBS for 45 min, rinsed three times with PBS for 5 min and permeabilized with 0.5% Triton X-100 in PBS for 5 min. Permeabilized cells were blocked in 5% BSA in PBS for 1 h. Cells were then incubated overnight at 4 °C with the following primary antibodies: guinea pig anti-MAP2 (Synaptic Systems, Goettingen, Germany; Cat # 188,004; RRID:AB_2138181) diluted 1:1000, rabbit anti-synaptophysin 1 (Synaptic Systems, Cat #: 101002; RRID:AB_887905) diluted 1:250 and mouse anti-PSD95 (ABCAM, Cambridge, UK, Cat #AB192757; RRID:AB_2750929) diluted 1:250. All dilutions were in 5% BSA in PBS. The following day, cultures were washed three times with PBS and incubated for 1 h at room temperature with secondary antibody, fluorescein goat anti-mouse IgG (Invitrogen, Thermo Fisher Scientific; Cat #A21131; RRID:AB_141618), goat anti-guinea pig AlexaFluor 568 (Invitrogen, Thermo Fisher Scientific; Cat #A11075; RRID:AB_141954) and goat anti-mouse IgG2a AlexaFluor 647 (Invitrogen, Thermo Fisher Scientific; Cat #A21241; RRID:AB_141698), all diluted 1: 1000 in 5% BSA in PBS. Slides were mounted in Invitrogen Prolong Gold Antifade Reagent with DAPI. Images of immunostained neurons were captured from at least twenty fields per well at 40x magnification, with three wells from four independent dissections per treatment per sex, using an automated high content imaging system (ImageXpress). Automated image analysis was performed using a cell scoring custom built journal in MetaXpress software (Molecular Devices; version 5.3.0.5) (Supplemental file 2, Fig S13).

### 2.8. Statistical and data analysis

Data are expressed as the mean ± SD. Sample size for all cultures was determined based on historical data (Hennebelle et al., 2020). The automated high content analysis was blinded for both treatment and sex. For axon outgrowth and Sholl analysis, means were determined based on a sample size of 8–9 from three independent dissections with coverslips serving as the unit of statistical measure (e.g., each coverslip was considered one biological replicate). For synaptic connectivity, means were determined based on a sample size of 10–12 from four independent dissections, with coverslips serving as the unit of statistical measure. For calcein AM and LDH release, means were determined based on a sample size of three, with three independent dissections as biological replicate. Statistical analysis was performed using GraphPad Prism 8 (La Jolla, CA, USA). All data were tested for normality using the Shapiro–Wilk test. One-way analysis of variance (ANOVA) was performed to analyze overall effects of treatment; male and female cultures were analyzed independently. Dunnett’s post-hoc test was used to identify specific treatment groups that differed significantly from vehicle controls. Unpaired student’s *t*-test was used to identify differences between technical controls and vehicle controls. Sholl profiles were analyzed by calculating the area under the curve (AUC) using built-in AUC analysis in GraphPad Prism Software, considering only peaks above the baseline. Comparison between male and female AUCs for each OXLAM was performed using unpaired Student’s t-test. P values ≤ 0.05 were considered significant. The data that support the findings of this study are available from the corresponding author upon reasonable request.

## 3. Results

### 3.1. Effect of OXLAMs on axonal morphogenesis

**Fig. 3.**
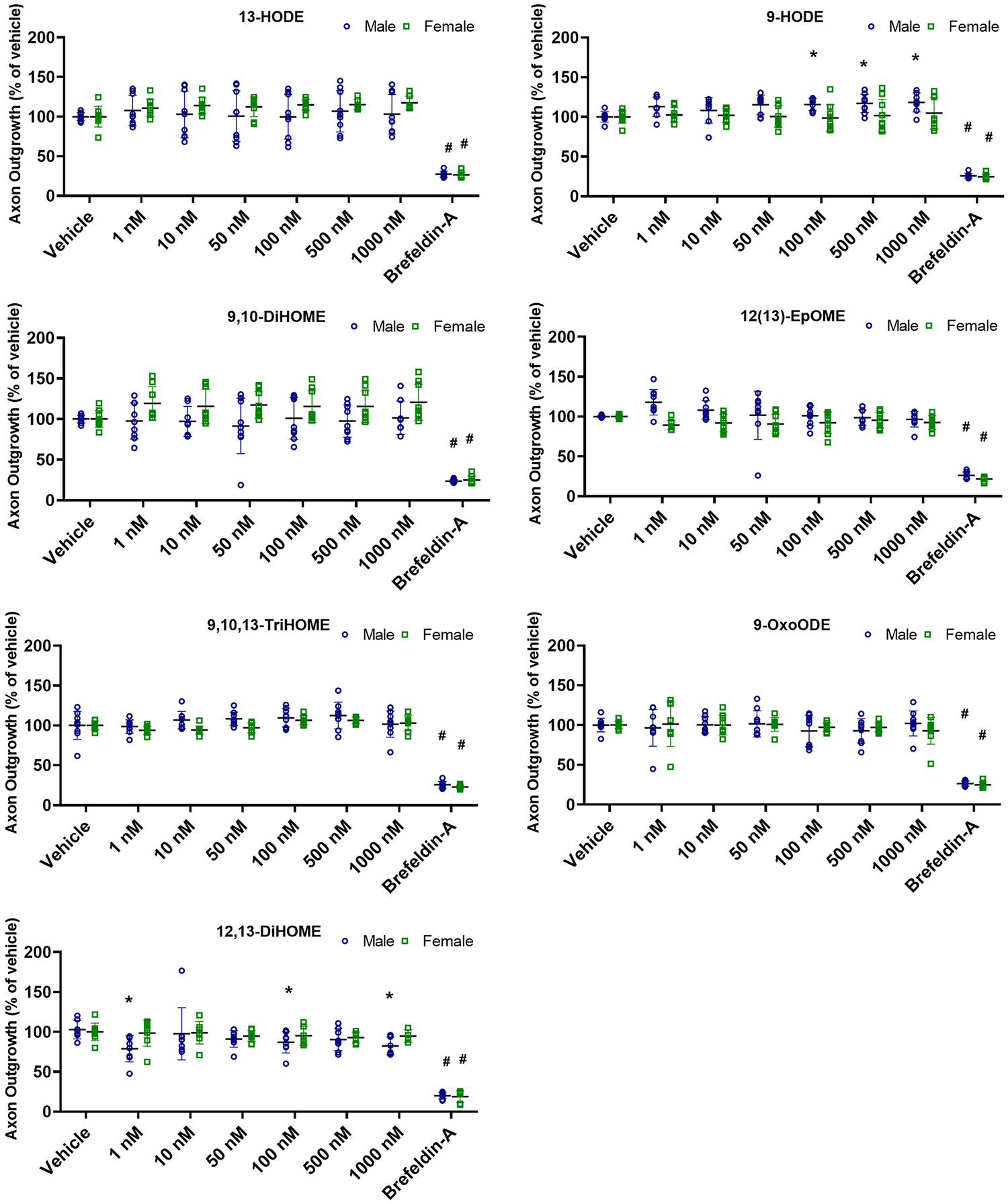
Effect of different concentrations of OXLAMS on axonal growth of primary rat cortical neurons. Axonal growth was quantified in DIV 2 cortical neurons following a 48 h exposure to varying concentrations of 13-HODE, 9-HODE, 9,10-DiHOME, 12(13)-EpOME, 9,10,13-TriHOME, 9-OxoODE or 12,13-DiHOME. Data shown in each panel represent the mean ± SD (n = 8–9 wells per treatment per sex from three independent dissections). *Significantly different from sex-matched vehicle control at p < 0.05 as determined using one-way ANOVA followed by Dunnett’s multiple comparison post hoc test. #Significantly different from vehicle control at p < 0.05 as determined using unpaired *t*-test.

**Fig. 4.**
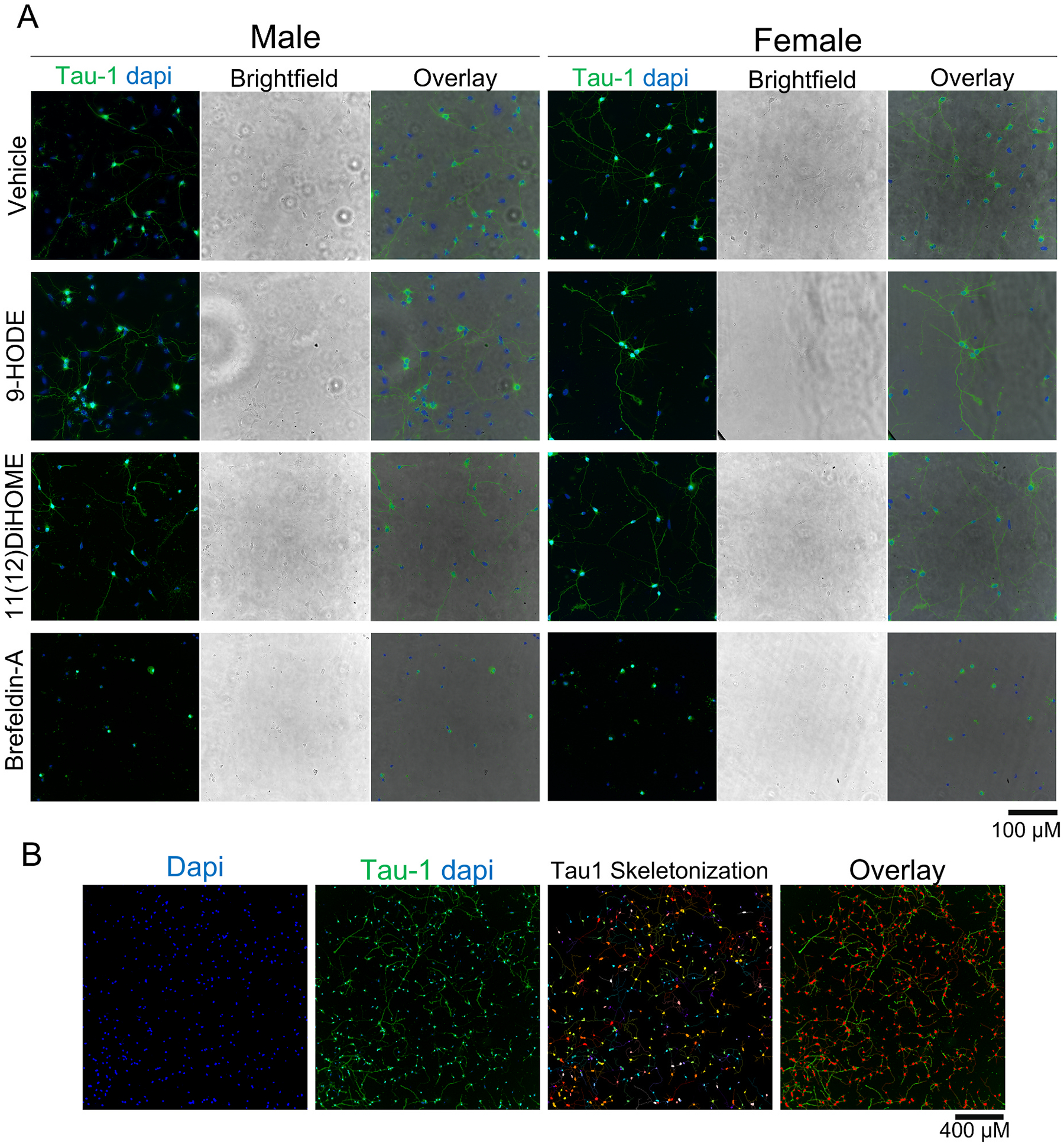
Representative photomicrographs of primary rat cortical neuron-glia co-cultures exposed to vehicle, 9-HODE at 1000 nM or 12,13-DiHOME at 1000 nM. Cultures were immunostained for Tau-1 at DIV 2 to quantify axonal growth. (A) Representative photomicrographs of images obtained at 40x on the ImageXpress high content imaging system. Scale bar = 100 μm. (B) Representative photomicrographs of a single field showing DAPI staining (blue), Tau-1 immunoreactivity (green) overlaid with DAPI staining, skeletonization mask of Tau-1 immunoreactivity, and overlay of Tau-1 with the skeletonization. Scale bar = 400 μm.

To evaluate the effects of OXLAMs on axonal morphogenesis, axonal length was quantified in DIV 2 neurons immunopositive for tau-1, a cytoskeletal protein localized to the axons of central neurons (Cleveland et al., 1977; Kanaan and Grabinski, 2021) following a 48 h exposure to an OXLAM or vehicle (0.1% ethanol). Relative to sex-matched vehicle control cultures, exposure to 9-HODE at 100, 500 or 1000 nM significantly increased the total axonal length of male, but not female, cortical neurons (Fig. 3). In contrast, exposure to 12,13-DiHOME at 1, 100 or 1000 nM significantly reduced axonal length in male cortical neurons but had no effect on axonal length in female cortical neurons. 13-HODE, 9-oxoODE, 9,10-DiHOME, 12(13)-EpOME and 9,10,13-TriHOME did not significantly alter axonal length in male or female cortical neurons at any of the concentrations tested (Fig. 3). These effects can be seen in representative photomicrographs of primary cortical neuron-glia co--cultures exposed to vehicle, 9-HODE at 1000 nM or 12,13-DiHOME at 1000 nM (Fig. 4).

### 3.2. Effect of OXLAMs on dendritic length and complexity

**Fig. 5.**
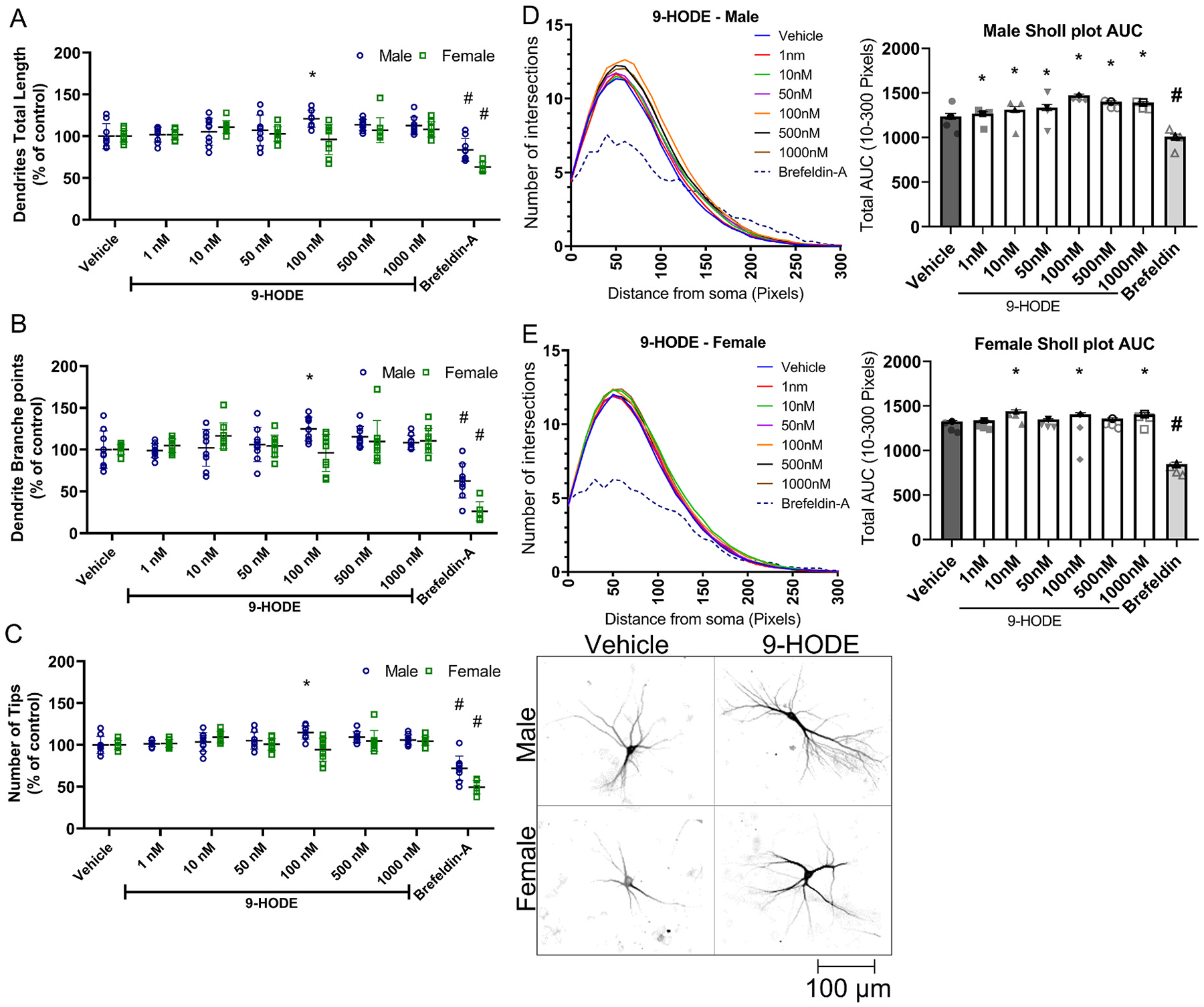
Effect of 9-HODE on the dendritic arborization of primary rat cortical neurons. Dendritic morphology was quantified in DIV 9 cortical neurons following a 48 h exposure to varying concentratons of 9-HODE. (A) Total dendritic length, (B) number of dendritic branch points, and (C) number of dendritic tips per neuron are plotted as the mean ± SD (n = 8–9 wells per treatment per sex from three independent dissections). (D,E) Sholl plots (left) showing the mean (n = 8–9 wells per treatment per sex from three independent dissections) number of dendritic intersections with Sholl rings at different distances from the cell body and bar graphs showing the mean ± SD (n = 8–9 wells per treatment per sex from three independent dissections) of the area under the curve (AUC) were determined independently for male (D) and female (E) cortical neurons. Data from individual neurons (40–80 neurons per well of 2–3 wells per dissection) were averaged within each independent dissection. *Significantly different from vehicle control at p < 0.05 as determined by one-way ANOVA followed by Dunnett’s multiple comparison post hoc test. #Significantly different from vehicle control at p < 0.05 as determined using unpaired *t*-test.

Sex-dependent significant changes in dendritic arborization were observed in cultures exposed to 9-HODE, 12(13)-EpOME and 9-OxoODE (Fig. 5), but not in cortical neurons of either sex exposed to any of the other OXLAMs that were tested (Supplemental file 1, Fig S2, S3, S4 and S5). One-way ANOVA followed by Dunnett’s multiple comparison revealed that relative to vehicle controls, 9-HODE at 100 nM enhanced total dendritic length, the number of dendritic branches and the number of total dendritic tips per neuron in male cortical neurons (Fig. 5 A, B, C). There were no significant effects of 9-HODE on the dendritic arborization of female cortical neurons. Sholl plots indicate that 9-HODE increased dendritic complexity, indicated as an increased AUC, in male cortical neurons at all concentrations tested, whereas in female cortical neurons, 9-HODE increased dendritic complexity only at 10, 100 and 1000 nM (Fig. 5 D, E).

**Fig. 6.**
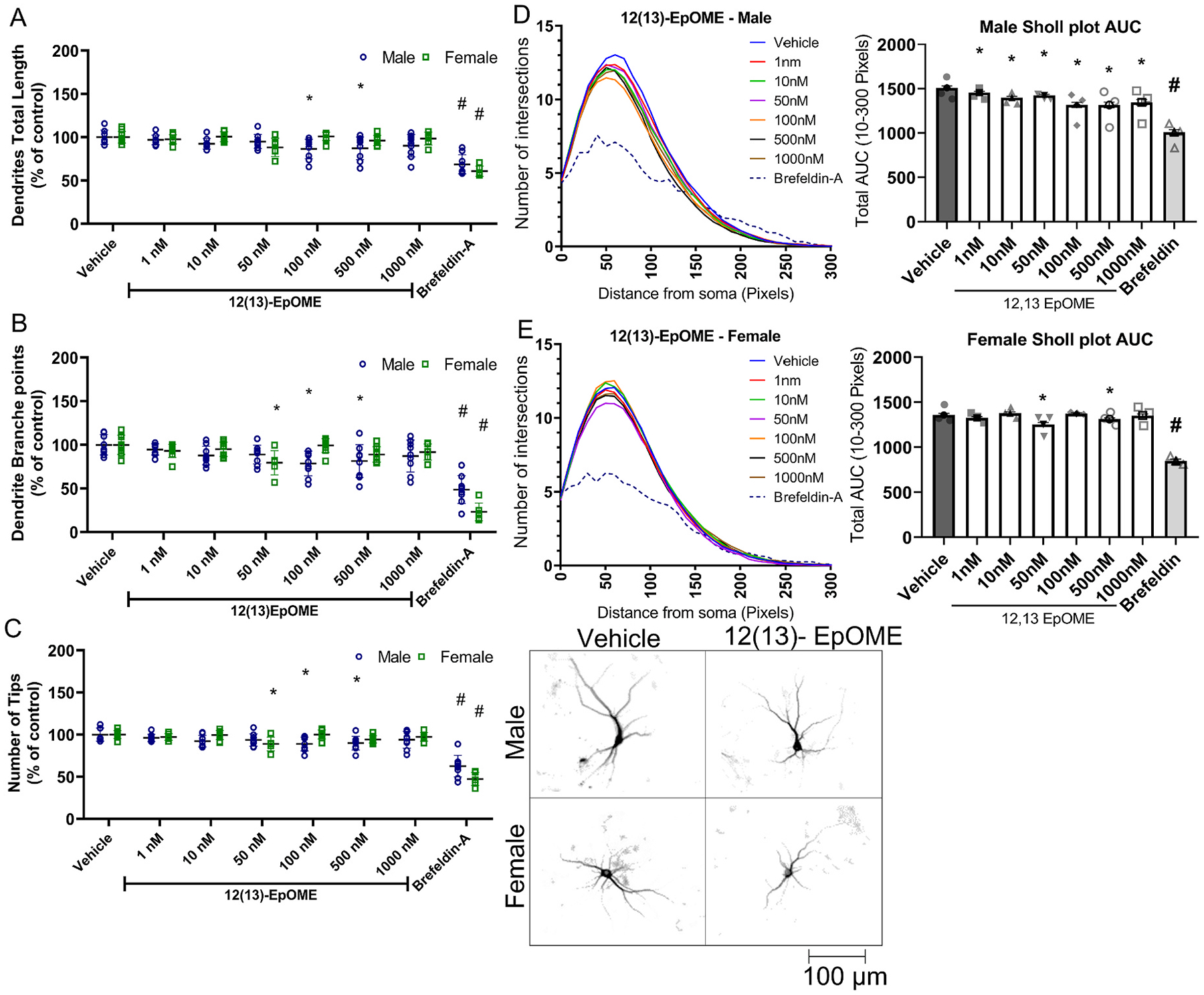
Effect of 12(13)-EpOME on the dendritic arborization of primary rat cortical neurons. Dendritic morphology was quantified in DIV 9 cortical neurons following a 48 h exposure to varying concentratons of 12(13)-EpOME. (A) Total dendritic length, (B) number of dendritic branch points, and (C) number of dendritic tips per neuron are plotted as the mean ± SD (n = 8–9 wells per treatment per sex from three independent dissections). (D,E) Sholl plots (left) showing the mean (n = 8–9 wells per treatment per sex from three independent dissections) number of dendritic intersections with Sholl rings at different distances from the cell body and bar graphs showing the mean ± SD (n = 8–9 wells per treatment per sex from three independent dissections) of the area under the curve (AUC) were determined independently for male (D) and female (E) cortical neurons. Data from individual neurons (40–80 neurons per well of 2–3 wells per dissection) were averaged within each independent dissection. *Significantly different from vehicle control at p < 0.05 as determined by one-way ANOVA followed by Dunnett’s multiple comparison post hoc test. #Significantly different from vehicle control at p < 0.05 as determined using unpaired *t*-test.

In contrast, 12(13)-EpOME significantly reduced dendritic arborization in male cortical neurons, as evidenced by significantly decreased total dendritic length, number of dendritic branches and number of total dendritic tips per neuron in cultures exposed to 12(13)-EpOME at 100 and 500 nM relative to sex-matched vehicle controls (Fig. 6 A, B, C). In female cortical neurons, 12(13)-EpOME at 50 nM significantly decreased the number of dendritic branches and total dendritic tips per neuron (Fig. 6 A, B, C). All concentrations of 12(13)-EpOME decreased the AUC of the Sholl plot in male cortical neurons relative to sex-matched vehicle controls. Female cortical neurons were significantly less affected by 12(13)-EpOME, with the AUC of the Sholl plot significantly decreased relative to sex-matched vehicle controls only in cultures exposed to 12(13)-EpOME at 50 or 500 nM (Fig. 6 D, E).

**Fig. 7.**
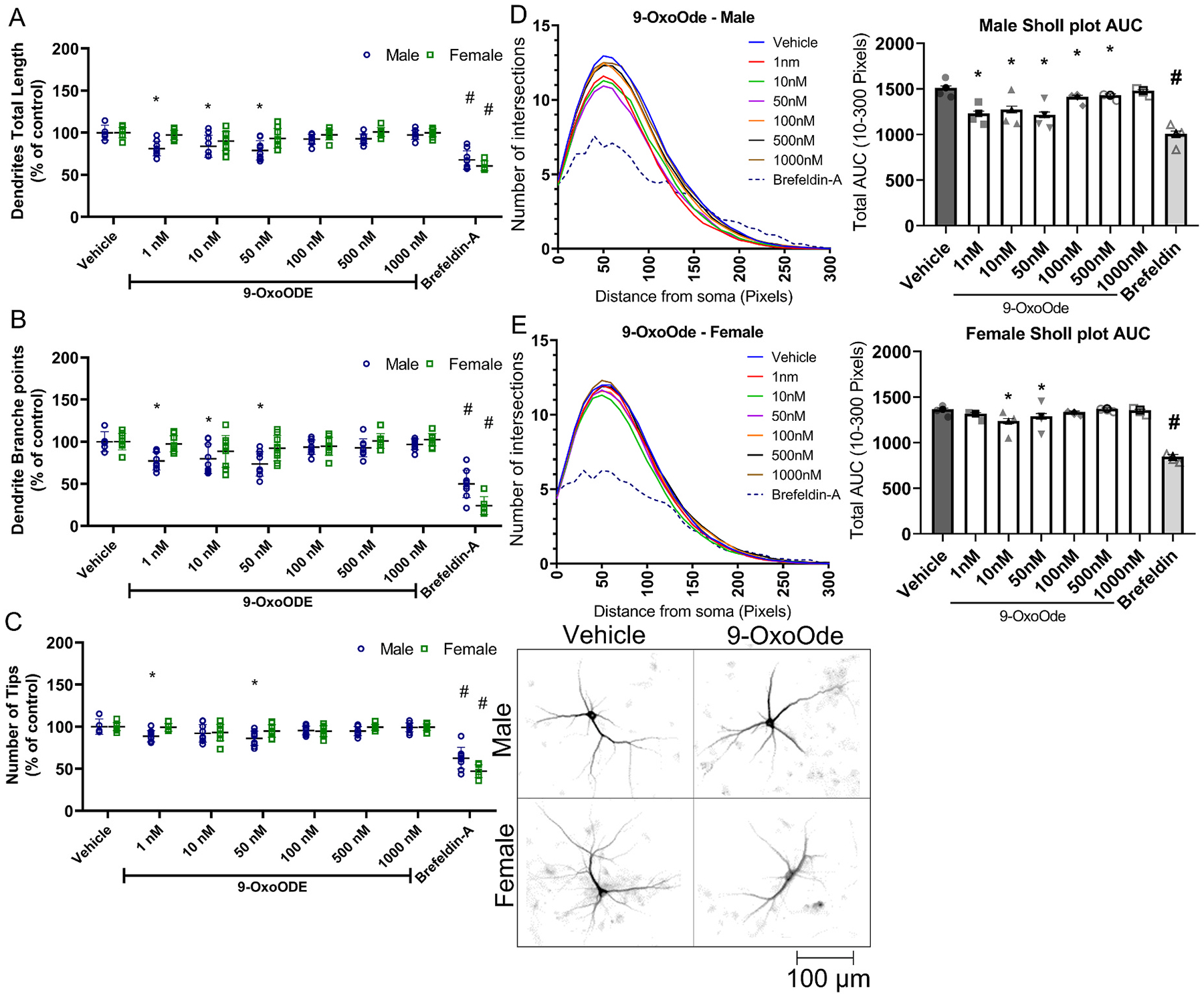
Effect of 9-OxoODE on the dendritic arborization of primary rat cortical neurons. Dendritic morphology was quantified in DIV 9 cortical neurons following a 48 h exposure to varying concentratons of 9-OxoODE. (A) Total dendritic length, (B) number of dendritic branch points, and (C) number of dendritic tips per neuron are plotted as the mean ± SD (n = 8–9 wells per treatment per sex from three independent dissections). (D,E) Sholl plots (left) showing the mean (n = 8–9 wells per treatment per sex from three independent dissections) number of dendritic intersections with Sholl rings at different distances from the cell body and bar graphs showing the mean ± SD (n = 8–9 wells per treatment per sex from three independent dissections) of the area under the curve (AUC) were determined independently for male (D) and female (E) cortical neurons. Data from individual neurons (40–80 neurons per well of 2–3 wells per dissection) were averaged within each independent dissection. *Significantly different from vehicle control at p < 0.05 as determined by one-way ANOVA followed by Dunnett’s multiple comparison post hoc test. #Significantly different from vehicle control at p < 0.05 as determined using unpaired *t*-test.

In male cortical neurons, exposure to 1, 10 or 50 nM of 9-OxoODE significantly decreased total dendritic length; in contrast, the number of dendritic branch points and total dendritic tips were decreased only by exposure to 9-OxoODE at 1 or 5 nM. The AUC of the Sholl plot was decreased by all concentrations of this OXLAM except 1000 nM (Fig. 7 A, B, C, D). Female cortical neurons were affected only by 9-OxoODE at 10 and 50 nM, as reflected by decreased AUC of the Sholl plot (Fig. 7 E).

**Fig. 8.**
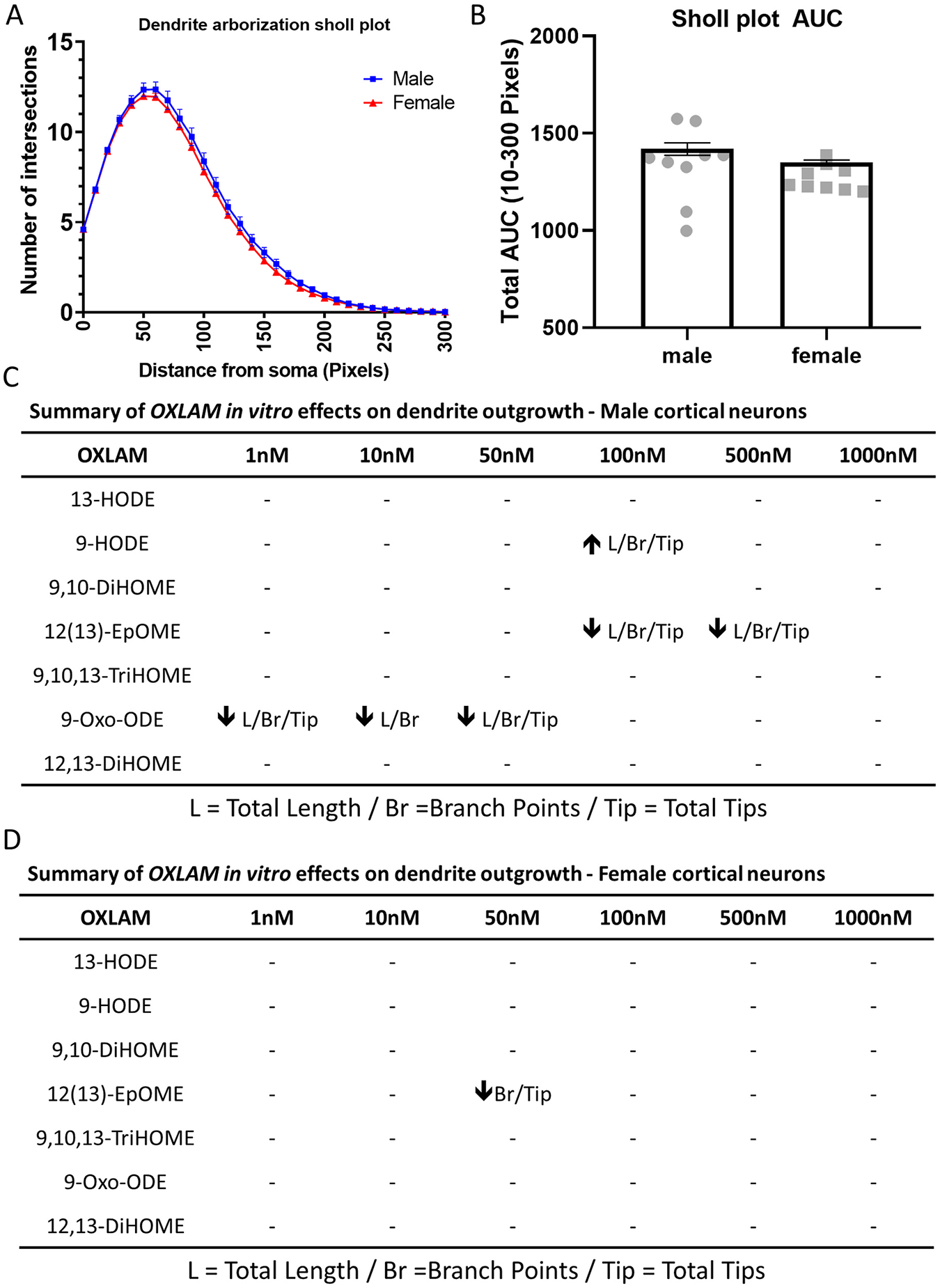
Summary of OXLAM effects on the dendritic arborization of primary rat cortical neurons. (A) Sholl plot comparing dendritic arborization of DIV 9 male and female cortical neurons cultured in the absence of OXLAMs. Data represent the mean ± SD (n = 8–9 wells per treatment per sex from three independent dissections) number of dendritic intersections with individual Sholl analysis ring spaced at varying distances from the neuronal cell body. Data from individual neurons (40–80 neurons per well of 2–3 wells per dissection) were averaged within each independent dissection. (B) Mean ± SD (n = 8–9 wells per treatment per sex from three independent dissections) area under the curve (AUC) in the Sholl plot. Unpaired *t*-test was used to compare the AUC for male versus female neurons. (C, D) Summary of the dendritic arborization data for each OXLAM test agent separated by sex. Up arrows indicate an increase compared to vehicle control; down arrows, a decrease compared to vehicle control; a hyphen (−) indicates not significantly different from vehicle control.

To account for any sex differences in dendritic arborization under basal conditions, Sholl analysis was conducted on untreated male and female cortical neurons. No significant difference in dendritic complexity between the sexes was found (Fig. 8 A, B).

### 3.3. Effect of OXLAMs on cell viability

To ensure that effects of OXLAMs on neuronal morphogenesis were not secondary to cytotoxic effects, cell viability was assessed following a 48 h exposure to OXLAMs at the same concentrations tested for effects on axonal and dendritic growth. Two independent tests of cell viability were applied: the release of lactate dehydrogenase (LDH) into the culture medium and uptake of calcein-AM and Hoechst-33342, which label live and dead cells, respectively. One-way ANOVA followed by Dunnett’s multiple comparison revealed that none of the OXLAMs altered LDH release compared to vehicle in either male or female neuron-glia co-cultures (Supplemental file 1, Fig S6, S7). Quantification of calcein-AM and Hoechst-33342 staining indicated that OXLAMs did not significantly alter the percentage of live cells relative to vehicle controls (Supplemental file 1, Fig S8, S9).

### 3.4. Effect of OXLAMs on synaptic connectivity

Synaptic connectivity was assessed by quantifying immunoreactivity for the presynaptic marker synaptophysin 1 (Wiedenmann and Franke, 1985), the postsynaptic marker PSD95 (Harrill et al., 2011; Koulen et al., 1998) and the dendrite-selective cytoskeletal protein MAP2B (Harrill et al., 2011, 2015). There were no significant effects of OXLAMs on these markers of synaptic connectivity compared to sex-matched vehicle controls in either male or female cortical cultures (Supplemental file 1, Fig S10).

## 4. Discussion

In the developing rat brain, OXLAMs account for approximately 50% of total oxidized fatty acids, and this is approximately 10 times higher than the 5–7% OXLAMs reported in adult rats (Hennebelle et al., 2020; Taha et al., 2018). This study demonstrated that at least some OXLAMs can modulate the morphogenesis of primary rat cortical neurons by altering the rate of axonal or dendritic growth. Of the 7 OXLAMs tested, only 9-HODE altered both dendritic and axonal growth, promoting the growth of both processes. In contrast, the other OXLAMs observed to alter neuronal morphogenesis had an inhibitory effect on processes growth, with 12,13-DiHOME decreasing axonal growth and 12 (13)-EpOME and 9-oxoODE reducing dendritic arborization. None of the OXLAMs tested in this study altered expression of synaptic markers, suggesting that OXLAMs do not influence synaptogenesis. We previously reported that 13-HODE, an OXLAM abundant in the brain, increased axonal outgrowth of male primary neuron-glia co-cultures at a concentration of 100 nM (Hennebelle et al., 2020). While we did not reproduce that finding in our present study, we did observe a trend towards increased axonal outgrowth in male and female cortical cultures exposed to 13-HODE. However, these differences did not reach statistical significance likely because of the normal biological variability between independent cultures and a lower effect size compared to other compounds in which significance was achieved (e.g. 9-HODE). This could also reflect the lower sample size of the present study (3 independent dissections) versus our previous study (4 independent dissections).

The concentration range of OXLAMs tested *in vitro* (1–1000 nM) encompasses previously reported brain levels *in vivo*. For example, considering a rat brain density of 1.04 g/mL (DiResta et al., 1990), we previously reported concentrations of 18.2 nM of 13-HODE, 13.2 nM of 9-HODE, 10.6 nM of 12(13)-EpOME, and 0.4–3.4 nM of the remaining OXLAMs in whole brain tissue from male rat pups (Hennebelle et al., 2020). While OXLAM concentrations within specific cell types within the brain are not known, they are likely to be greater than whole brain levels since the latter includes multiple cell types, non-cellular structures and water. Further complicating comparison of *in vitro* concentrations to *in vivo* levels is the fact that bovine serum albumin, a component of our tissue culture medium that would not be found in the brain parenchyma, has been shown to bind free oxylipins (Maddipati and Zhou, 2011), thereby effectively decreasing the concentrations available to cultured neurons. Other studies that tested the *in vitro* effects of OXLAMs have used concentrations similar to those used here. For instance, a study which tested 9-HODE-mediated activation of G2A receptors showed effects at concentrations greater than 300 nM (Obinata et al., 2005). Another study that looked at PPARγ ligand displacement by OXLAMs used an IC50 of 10 μM for 9-HODE, and above 0.8 μM (800 nM) for 9(10) EpOME (Lecka-Czernik et al., 2002).

The pharmacodynamic effect of OXLAMs on axonal and dendritic morphology exhibited a non-monotonic concentration-effect relationship as shown in the Sholl plots (Figs. 3–5 - D and E). Non-monotonic dose-related effects are not uncommon in both *in vivo* and *in vitro* studies (extensively reviewed by Vandenberg et al., 2012; Zoeller and Vandenberg, 2015). For instance, a similar inverted U-shaped dose-response was previously described for neuron-glia co-cultures treated with 13-HODE (Hennebelle et al., 2020) or with polychlorinated biphenyls (Keil et al., 2018; Yang et al., 2014). Mechanisms underlying this non-monotonic response to the morphogenic activity of OXLAMs are yet to be determined; however, the lack of OXLAM toxicity at all concentrations tested in the current study suggests that the differential responses observed at higher vs. lower concentrations are not due to cytotoxicity at the higher concentrations.

The mechanism(s) underlying the regulation of neuronal morphogenesis by OXLAMs remain speculative; however, none of the OXLAMs altered cell viability, suggesting that OXLAM effects on axonal and dendritic growth were not simply a non-specific consequence of cytotoxicity. While we cannot rule out the possibility that the axon- and dendrite-promoting activity of 9-HODE occur as a result of general hypertrophic effects of this OXLAM, the observation that other OXLAMs differentially affected axons vs. dendrites suggests these compounds influence neuronal morphogenesis via selective modulation of molecular processes that uniquely control axonal versus dendritic growth.

9-HODE is an agonist of PPARγ (Nagy et al., 1998; Schild et al., 2002), which when activated stimulates axonal outgrowth in primary rat hippocampal neurons and human neuroblastoma cells (Inestrosa et al., 2005; Miglio et al., 2009). 9-OxoODE, a ketone metabolite of 9-HODE (Kühn et al., 1990), has the opposite effect on dendritic morphology. While the reason(s) for these opposing effects are not known, it is possible that 9-HODE and 9-oxoODE act through different G-protein coupled receptors in the brain. 9-HODE is known to increase intracellular calcium signaling through the G2A receptor, although other receptors are likely involved since binding to G2A is non-specific (i.e. other oxylipins also bind to the same receptor with similar affinities) (Obinata et al., 2005). To our knowledge, specific-protein receptors for 9-oxoODE have not been characterized; however, it is not unusual for lipid mediator analogues to have opposing effects in biological systems. For example, arachidonic acid-derived prostaglandin E2 is known to promote inflammation *in vitro* and *in vivo* (Goulet et al., 2004), whereas its oxidized metabolite, prostaglandin D2, is thought to have anti-inflammatory effects (Ajuebor et al., 2000). 12,13-DiHOME decreased the axonal length of male cortical neurons and has been shown to induce mitochondrial dysfunction (Moran et al., 2001; Sisemore et al., 2001), and to cause transient receptor potential vanilloid 1 (TRPV1)-dependent calcium influx in mice dorsal root ganglion neurons (Zimmer et al., 2018). Whether these mechanisms mediate the axon inhibitory activity of 12,13-DiHOME remains to be determined.

Findings from this study also suggest that the morphogenic effects of OXLAMs are sex specific (see summary in Fig. 8 C and D). In support of this conclusion, we observed that: (1) 9-HODE increased axonal growth while 12(13)-DiHOME decreased axonal growth in male but not female cortical neurons; (2) the dendritic effects of 9-HODE and 9-OxoODE were specific to male cortical neurons; and (3) while 12(13)-EpOME altered dendritic arborization in both male and female cortical neurons, male cortical neurons appeared to be more sensitive. The third observation derives from the finding that (1) the total dendritic length, number of dendritic branch points, number of dendritic tips and AUC were altered in male cortical neurons, but only AUC was affected in female cortical neurons; and (2) the AUC was significantly reduced at lower concentrations of 12(13)-EpOME in male cortical cultures (1 nM) compared to female cortical cultures (50 nM).

The sex-dependent effects of OXLAMs on neuronal morphogenesis may reflect sex differences in the rate of neuronal maturation. For example, it has been reported that the dendritic arborization of hippocampal neurons in neuron-glia co-cultures derived from male mouse pups is significantly greater than that of hippocampal neurons from female littermates (Keil et al., 2017). However, that same study found that there were no sex differences in the dendritic arborization of cortical neurons cultured from these same animals (Keil et al., 2017). Similarly, we observed in this study that the dendritic arborization of cortical neurons in neuron-glia co-cultures derived from rat pups did not differ between males and females. Collectively, these observations suggest that the sex-specific effects of OXLAMs on neuronal morphogenesis are likely due to male neurons being more sensitive to modulation by OXLAMs than females.

Findings from this study raise the possibility that OXLAMs regulate neuronal morphogenesis *in vivo*. If true, then elevated infant exposure to OXLAMs *in utero* or postnatally (through breast milk) may lead to long-lasting changes in neuronal connectivity and behavior. *In vivo*, OXLAMs have been shown to regulate the brain’s response to ischemic injury and to peripheral pain sensitization (Hennebelle et al., 2017; Jensen et al., 2018). While these observations support a bioactive role of OXLAMs in the adult rat brain, no studies have yet tested whether OXLAMs influence the brain during early development when OXLAMs have been shown to constitute the majority (>50%) of oxylipins in the brain (Hennebelle et al., 2020). However, there are extensive clinical and experimental animal data demonstrating that altered spatiotemporal patterns of axonal and dendritic growth are associated with neurodevelopmental disorders in humans (Copf, 2016; Engle, 2010; Garey, 2010; Penzes et al., 2011; Robichaux and Cowan, 2014; Supekar et al., 2013) and behavioral phenotypes in animal models (Berger-Sweeney and Hohmann, 1997; Cremer et al., 1997; Maier et al., 1999). Future studies are needed to understand the developmental implications of increased exposure to OXLAMs in early life.

## 5. Conclusions

Our data demonstrate that OXLAMs modulate the morphogenesis of cortical neurons derived from the neocortex of neonatal Sprague Dawley rats in a sex-dependent manner. These novel findings suggest a previously unknown biological role of OXLAMs in regulating neurodevelopmental processes critical to establishing neural circuits in the developing brain. Given that altered patterns of neuronal connectivity, including increased and decreased axonal growth and dendritic arborization, are thought to be the biological substrate of many neurodevelopmental disorders (Belichenko et al., 2009; Gilmore et al., 2004; Jiang et al., 2013; Mainen and Sejnowski, 1996), and that a number of neurodevelopmental disorders differentially affect males versus females (May et al., 2019), these finding have potential implications for understanding the etiology and sex specificity of neurodevelopmental disorders.

## Supplementary Material

supplemental file 1

supplemental file 2

## Data Availability

Data will be made available on request.

## Abbreviations

12(13)-EpOME: 12(13)-epoxyoctadecenoic acid
12,13-DiHOME: 12,13-dihydroxyoctadecenoic acid
13-HODE: 13-hydroxyoctadecadienoic acid
9,10,13-TriHOME: 9,10,13-trihydroxyoctadecenoic acid
9,10-DiHOME: 9,10-dihydroxyoctadecenoic acid
9-HODE: 9-hydroxyoctadecadienoic acid
9-OxoODE: 9-oxo-octadecadienoic acid
ALA: alpha-linolenic acid
AraC: cytosine-arabinoside
AUC: area under the curve
BSA: bovine serum albumin
COX: cyclooxygenase
CYP450: cytochrome P450
DIV: Day in vitro
LDH: lactate dehydrogenase
LA: linoleic acid
LOX: lipoxygenase
MAP2B: Microtubule-associated protein 2B
Min: Minutes
OXLAMs: oxidized linoleic acid metabolites
PB: phosphate-buffer
PUFAs: polyunsaturated fatty acids
sEH: soluble epoxide hydrolase (sEH)

## References

[R1] Ajuebor MN, Singh A, Wallace JL, 2000. Cyclooxygenase-2-derived prostaglandin D (2) is an early anti-inflammatory signal in experimental colitis. Am. J. Physiol. Gastrointest. Liver Physiol 279 (1), G238–G244. 10.1152/ajpgi.2000.279.1.G238. 10898767

[R2] Belichenko PV, Wright EE, Belichenko NP, Masliah E, Li HH, Mobley WC, Francke U, 2009. Widespread changes in dendritic and axonal morphology in Mecp2-mutant mouse models of Rett syndrome: evidence for disruption of neuronal networks. J. Comp. Neurol 514 (3), 240–258. 10.1002/cne.22009. 19296534

[R3] Berger-Sweeney J, Hohmann CF, 1997. Behavioral consequences of abnormal cortical development: insights into developmental disabilities. Behav. Brain Res 86 (2), 121–142. 9134147 10.1016/s0166-4328(96)02251-6

[R4] Bernard JY, Armand M, Garcia C, Forhan A, De Agostini M, Charles M-A, Heude B, 2015. The association between linoleic acid levels in colostrum and child cognition at 2 and 3 y in the EDEN cohort. Pediatr. Res 77 (6), 829–835. 10.1038/pr.2015.50. 25760551

[R5] Blasbalg TL, Hibbeln JR, Ramsden CE, Majchrzak SF, Rawlings RR, 2011. Changes in consumption of omega-3 and omega-6 fatty acids in the United States during the 20th century. Am. J. Clin. Nutr 93 (5), 950–962. 10.3945/ajcn.110.006643. 21367944 PMC3076650

[R6] Cleveland DW, Hwo SY, Kirschner MW, 1977. Purification of tau, a microtubule-associated protein that induces assembly of microtubules from purified tubulin. J. Mol. Biol 116 (2), 207–225. 10.1016/0022-2836(77)90213-3. 599557

[R7] Copf T, 2016. Impairments in dendrite morphogenesis as etiology for neurodevelopmental disorders and implications for therapeutic treatments. Neurosci. Biobehav. Rev 68, 946–978. 10.1016/j.neubiorev.2016.04.008. 27143622

[R8] Cremer H, Chazal G, Goridis C, Represa A, 1997. NCAM is essential for axonal growth and fasciculation in the hippocampus. Mol. Cell. Neurosci 8 (5), 323–335. 10.1006/mcne.1996.0588. 9073395

[R9] Cunnane SC, Williams SC, Bell JD, Brookes S, Craig K, Iles RA, Crawford MA, 1994. Utilization of uniformly labeled 13C-polyunsaturated fatty acids in the synthesis of long-chain fatty acids and cholesterol accumulating in the neonatal rat brain. J. Neurochem 62 (6), 2429–2436. 10.1046/j.1471-4159.1994.62062429.x. 8189246

[R10] DeMar JC, Lee HJ, Ma K, Chang L, Bell JM, Rapoport SI, Bazinet RP, 2006. Brain elongation of linoleic acid is a negligible source of the arachidonate in brain phospholipids of adult rats. Biochim. Biophys. Acta, Mol. Cell Biol. Lipids 1761 (9), 1050–1059. 10.1016/j.bbalip.2006.06.006. 16920015

[R11] DiResta GR, Lee J, Lau N, Ali F, Galicich JH, Arbit E, 1990. Measurement of brain tissue density using pycnometry. Acta Neurochir. Suppl 51, 34–36. 10.1007/978-3-7091-9115-6_12. 2089932

[R12] Earles SM, Bronstein JC, Winner DL, Bull AW, 1991. Metabolism of oxidized linoleic acid: characterization of 13-hydroxyoctadecadienoic acid dehydrogenase activity from rat colonic tissue. Biochim. Biophys. Acta Lipids Lipid. Metabol 1081 (2), 174–180. 10.1016/0005-2760(91)90023-B. 1998735

[R13] Engle EC, 2010. Human genetic disorders of axon guidance. Cold Spring Harbor Perspect. Biol 2 (3), a001784 10.1101/cshperspect.a001784. PMC2829956 20300212

[R14] Funk CD, Powell WS, 1983. Metabolism of linoleic acid by prostaglandin endoperoxide synthase from adult and fetal blood vessels. Biochim. Biophys. Acta 754 (1), 57–71. 10.1016/0005-2760(83)90082-6. 6414520

[R15] Garey L, 2010. When cortical development goes wrong: schizophrenia as a neurodevelopmental disease of microcircuits. J. Anat 217 (4), 324–333. 10.1111/j.1469-7580.2010.01231.x. 20408906 PMC2992411

[R16] Gibson RA, Kneebone GM, 1981. Fatty acid composition of human colostrum and mature breast milk. Am. J. Clin. Nutr 34 (2), 252–257. 10.1093/ajcn/34.2.252. 7211726

[R17] Gilmore JH, Jarskog LF, Vadlamudi S, Lauder JM, 2004. Prenatal infection and risk for schizophrenia: IL-1β, IL-6, and TNFα inhibit cortical neuron dendrite development. Neuropsychopharmacology 29 (7), 1221–1229. 10.1038/sj.npp.1300446. 15085088

[R18] Goulet JL, Pace AJ, Key ML, Byrum RS, Nguyen M, Tilley SL, Morham SG, Langenbach R, Stock JL, McNeish JD, Smithies O, Coffman TM, Koller BH, 2004. E-Prostanoid-3 receptors mediate the proinflammatory actions of prostaglandin E 2 in acute cutaneous inflammation. J. Immunol 173 (2), 1321–1326. 10.4049/jimmunol.173.2.1321. 15240726

[R19] Green P, Yavin E, 1993. Elongation, desaturation, and esterification of essential fatty acids by fetal rat brain in vivo. JLR (J. Lipid Res.) 34 (12), 2099–2107. http://www.ncbi.nlm.nih.gov/pubmed/7905509. 7905509

[R20] Hansen AE, Haggard ME, Boelsche AN, Adam DJD, Wiese HF, 1958. Essential fatty acids in infant nutrition. J. Nutr 66 (4), 565–576. 10.1093/jn/66.4.565. 13621281

[R21] Harrill JA, Chen H, Streifel KM, Yang D, Mundy WR, Lein PJ, 2015. Ontogeny of biochemical, morphological and functional parameters of synaptogenesis in primary cultures of rat hippocampal and cortical neurons. Mol. Brain 8 (1). 10.1186/s13041-015-0099-9. PMC4339650 25757474

[R22] Harrill JA, Robinette BL, Mundy WR, 2011. Use of high content image analysis to detect chemical-induced changes in synaptogenesis in vitro. Toxicol. Vitro 25 (1), 368–387. 10.1016/j.tiv.2010.10.011. 20969947

[R23] Hassam AG, Sinclair AJ, Cramwford MA, 1975. The incorporation of orally fed radioactive γ-linolenic acid and linoleic acid into the liver and brain lipids of suckling rats. Lipids 10 (7), 417–420. 10.1007/BF02532447. 27519251

[R24] Hennebelle M, Metherel AH, Kitson AP, Otoki Y, Yang J, Lee KSS, Hammock BD, Bazinet RP, Taha AY, 2019. Brain oxylipin concentrations following hypercapnia/ischemia: effects of brain dissection and dissection time. JLR (J. Lipid Res.) 60 (3), 671–682. 10.1194/jlr.D084228. PMC6399504 30463986

[R25] Hennebelle M, Morgan RK, Sethi S, Zhang Z, Chen H, Grodzki AC, Lein PJ, Taha AY, 2020. Linoleic acid-derived metabolites constitute the majority of oxylipins in the rat pup brain and stimulate axonal growth in primary rat cortical neuron-glia co-cultures in a sex-dependent manner. J. Neurochem 152 (2), 195–207. 10.1111/jnc.14818. 31283837 PMC6949423

[R26] Hennebelle M, Zhang Z, Metherel AH, Kitson AP, Otoki Y, Richardson CE, Yang J, Lee KSS, Hammock BD, Zhang L, Bazinet RP, Taha AY, 2017. Linoleic acid participates in the response to ischemic brain injury through oxidized metabolites that regulate neurotransmission. Sci. Rep 7 (1), 1–14. 10.1038/s41598-017-02914-7. 28659576 PMC5489485

[R27] Inestrosa NC, Godoy JA, Quintanilla RA, Koenig CS, Bronfman M, 2005. Peroxisome proliferator-activated receptor γ is expressed in hippocampal neurons and its activation prevents β-amyloid neurodegeneration: role of Wnt signaling. Exp. Cell Res 304 (1), 91–104. 10.1016/j.yexcr.2004.09.032. 15707577

[R28] Jareb M, Banker G, 1997. Inhibition of axonal growth by brefeldin A in hippocampal neurons in culture. J. Neurosci 17 (23), 8955–8963. 10.1523/jneurosci.17-23-08955.1997. 9364043 PMC6573598

[R29] Jensen JR, Pitcher MH, Yuan ZX, Ramsden CE, Domenichiello AF, 2018. Concentrations of oxidized linoleic acid derived lipid mediators in the amygdala and periaqueductal grey are reduced in a mouse model of chronic inflammatory pain. Prostaglandins Leukot. Essent. Fatty Acids 135 (July), 128–136. 10.1016/j.plefa.2018.07.015. 30103924 PMC6269101

[R30] Jensen RG, Ferris AM, Lammi-Keefe CJ, 1992. Lipids in human milk and infant formulas. Annu. Rev. Nutr 12, 417–441. 10.1146/annurev.nu.12.070192.002221. 1503813

[R31] Jiang M, Ash RT, Baker SA, Suter B, Ferguson A, Park J, Rudy J, Torsky SP, Chao HT, Zoghbi HY, Smirnakis SM, 2013. Dendritic arborization and spine dynamics are abnormal in the mouse model of MECP2 duplication syndrome. J. Neurosci 33 (50), 19518–19533. 10.1523/JNEUROSCI.1745-13.2013. 24336718 PMC3858623

[R32] Kanaan NM, Grabinski T, 2021. Neuronal and glial distribution of tau protein in the adult rat and monkey. Front. Mol. Neurosci 14 (April), 1–27. 10.3389/fnmol.2021.607303. PMC8112591 33986642

[R33] Keil KP, Miller GW, Chen H, Sethi S, Schmuck MR, Dhakal K, Kim JW, Lein PJ, 2018. PCB 95 promotes dendritic growth in primary rat hippocampal neurons via mTOR-dependent mechanisms. Arch. Toxicol 92 (10), 3163–3173. 10.1007/s00204-018-2285-x. 30132043 PMC6162988

[R34] Keil KP, Sethi S, Wilson MD, Chen H, Lein PJ, 2017. In vivo and in vitro sex differences in the dendritic morphology of developing murine hippocampal and cortical neurons. Sci. Rep 7 (1), 1–15. 10.1038/s41598-017-08459-z. 28814778 PMC5559594

[R35] Koulen P, Fletcher EL, Craven SE, Bredt DS, Wassle H, 1998. Immunocytochemical localization of the postsynaptic density protein PSD- 95 in the mammalian retina. J. Neurosci 18 (23), 10136–10149. 10.1523/jneurosci.18-23-10136.1998. 9822767 PMC6793313

[R36] Kühn H, Belkner J, Wiesner R, Alder L, 1990. Occurrence of 9- and 13-keto-octadecadienoic acid in biological membranes oxygenated by the reticulocyte lipoxygenase. Arch. Biochem. Biophys 279 (2), 218–224. 10.1016/0003-9861(90)90484-G. 2112367

[R37] Laneuville O, Breuer DK, Xu N, Huang ZH, Gage DA, Watson JT, Lagarde M, DeWitt DL, Smith WL, 1995. Fatty acid substrate specificities of human prostaglandin-endoperoxide H synthase-1 and − 2. J. Biol. Chem 270 (33), 19330–19336. 10.1074/jbc.270.33.19330. 7642610

[R38] Lecka-Czernik B, Moerman EJ, Grant DF, Lehmann JM, Manolagas SC, Jilka RL, 2002. Divergent effects of selective peroxisome proliferator-activated receptor-gamma 2 ligands on adipocyte versus osteoblast differentiation. Endocrinology 143 (6), 2376–2384. 10.1210/endo.143.6.8834. 12021203

[R39] Lesslich HM, Klapal L, Wilke J, Haak A, Dietzel ID, 2022. Adjusting the neuron to astrocyte ratio with cytostatics in hippocampal cell cultures from postnatal rats: a comparison of cytarabino furanoside (AraC) and 5-fluoro-2’-deoxyuridine (FUdR). PLoS One 17 (3 March), 1–16. 10.1371/journal.pone.0265084. PMC8906639 35263366

[R40] Lien EL, Boyle FG, Yuhas RJ, Kuhlman CF, 1994. Effect of maternal dietary arachidonic or linoleic acid on rat pup fatty acid profiles. Lipids 29 (1), 53–59. 10.1007/BF02537091. 8139396

[R41] Maddipati KR, Zhou SL, 2011. Stability and analysis of eicosanoids and docosanoids in tissue culture media. Prostag. Other Lipid Mediat 94 (1–2), 59–72. 10.1016/j.prostaglandins.2011.01.003. 21236355

[R42] Maier DL, Mani S, Donovan SL, Soppet D, Tessarollo L, McCasland JS, Meiri KF, 1999. Disrupted cortical map and absence of cortical barrels in growth-associated protein (GAP)-43 knockout mice. Proc. Natl. Acad. Sci. U. S. A 96 (16), 9397–9402. 10430954 10.1073/pnas.96.16.9397 PMC17794

[R43] Mainen ZF, Sejnowski TJ, 1996. Influence of dendritic structure on firing pattern in model neocortical neurons. Nature 382 (6589), 363–366. 10.1038/382363a0. 8684467

[R44] Martínez M, Mougan I, 2002. Fatty acid composition of human brain phospholipids during normal development. J. Neurochem 71 (6), 2528–2533. 10.1046/j.1471-4159.1998.71062528.x. 9832152

[R45] May T, Adesina I, McGillivray J, Rinehart NJ, 2019. Sex differences in neurodevelopmental disorders. Curr. Opin. Neurol 32 (4), 622–626. 10.1097/WCO.0000000000000714. 31135460

[R46] Mendonca MA, Araujo WMC, Borgo LA, Alencar EDR, 2017. Lipid profile of different infant formulas for infants. PLoS One 12 (6), 1–14. 10.1371/journal.pone.0177812. PMC5453432 28570611

[R47] Miglio G, Rattazzi L, Rosa AC, Fantozzi R, 2009. PPARγ stimulation promotes neurite outgrowth in SH-SY5Y human neuroblastoma cells. Neurosci. Lett 454 (2), 134–138. 10.1016/j.neulet.2009.03.014. 19429070

[R48] Moran JH, Nowak G, Grant DF, 2001. Analysis of the toxic effects of linoleic acid, 12, 13-cis-epoxyoctadecenoic acid, and 12,13-dihydroxyoctadecenoic acid in rabbit renal cortical mitochondria. Toxicol. Appl. Pharmacol 172 (2), 150–161. 10.1006/taap.2001.9149. 11298501

[R49] Nagy L, Tontonoz P, Alvarez JGA, Chen H, Evans RM, 1998. Oxidized LDL regulates macrophage gene expression through ligand activation of PPARγ. Cell 93 (2), 229–240. 10.1016/S0092-8674(00)81574-3. 9568715

[R50] Obinata H, Hattori T, Nakane S, Tatei K, Izumi T, 2005. Identification of 9-hydroxyoctadecadienoic acid and other oxidized free fatty acids as ligands of the G protein-coupled receptor G2A. J. Biol. Chem 280 (49), 40676–40683. 10.1074/jbc.M507787200. 16236715

[R51] Penzes P, Cahill ME, Jones KA, VanLeeuwen JE, Woolfrey KM, 2011. Dendritic spine pathology in neuropsychiatric disorders. Nat. Neurosci 14 (3), 285–293. 10.1038/nn.2741. 21346746 PMC3530413

[R52] Putnam JC, Carlson SE, DeVoe PW, Barness LA, 1982. The effect of variations in dietary fatty acids on the fatty acid composition of erythrocyte phosphatidylcholine and phosphatidylethanolamine in human infants. Am. J. Clin. Nutr 36 (1), 106–114. 10.1093/ajcn/36.1.106. 7091020

[R53] Reinaud O, Delaforge M, Boucher JL, Rocchiccioli F, Mansuy D, 1989. Oxidative metabolism of linoleic acid by human leukocytes. Biochem. Biophys. Res. Commun 161 (2), 883–891. 10.1016/0006-291x(89)92682-x. 2735926

[R54] Robichaux MA, Cowan CW, 2014. Signaling mechanisms of axon guidance and early synaptogenesis. Current Topics in Behavioral Neuroscience 16, 19–48. 10.1007/7854_2013_255. 24318963

[R55] Sanders TAB, Mistry M, Naismith DJ, 1984. The influence of a maternal diet rich in linoleic acid on brain and retinal docosahexaenoic acid in the rat. Br. J. Nutr 51 (1), 57–66. 10.1079/bjn19840009. 6228249

[R56] Schild RL, Schaiff WT, Carlson MG, Cronbach EJ, Nelson DM, Sadovsky Y, 2002. The activity of PPARγin primary human trophoblasts is enhanced by oxidized lipids. J. Clin. Endocrinol. Metab 87 (3), 1105–1110. 10.1210/jc.87.3.1105. 11889173

[R57] Schmuck MR, Keil KP, Sethi S, Morgan RK, Lein PJ, 2020. Automated high content image analysis of dendritic arborization in primary mouse hippocampal and rat cortical neurons in culture. J. Neurosci. Methods 341 (April), 108793. 10.1016/j.jneumeth.2020.108793. 32461071 PMC7357201

[R58] Sethi S, Keil KP, Lein PJ, 2017. Species and sex differences in the morphogenic response of primary rodent neurons to 3,3′-dichlorobiphenyl (PCB 11). Toxics 6 (1). 10.3390/toxics6010004. PMC5874777 29295518

[R59] Sisemore MF, Zheng J, Yang JC, Thompson DA, Plopper CG, Cortopassi GA, Hammock BD, 2001. Cellular characterization of leukotoxin diol-induced mitochondrial dysfunction. Arch. Biochem. Biophys 392 (1), 32–37. 10.1006/abbi.2001.2434. 11469791

[R60] Supekar K, Uddin LQ, Khouzam A, Phillips J, Gaillard WD, Kenworthy LE, Yerys BE, Vaidya CJ, Menon V, 2013. Brain hyperconnectivity in children with autism and its links to social deficits. Cell Rep 5 (3), 738–747. 10.1016/j.celrep.2013.10.001. 24210821 PMC3894787

[R61] Taha AY, Hennebelle M, Yang J, Zamora D, Rapoport SI, Hammock BD, Ramsden CE, 2018. Regulation of rat plasma and cerebral cortex oxylipin concentrations with increasing levels of dietary linoleic acid. Prostagl. Leukot. Essent. Fat. Acids 138, 71–80. 10.1016/j.plefa.2016.05.004. PMC5106341 27282298

[R62] Vandenberg LN, Colborn T, Hayes TB, Heindel JJ, Jacobs DR, Lee DH, Shioda T, Soto AM, vom Saal FS, Welshons WV, Zoeller RT, Myers JP, 2012. Hormones and endocrine-disrupting chemicals: low-dose effects and nonmonotonic dose responses. Endocr. Rev 33 (3), 378–455. 10.1210/er.2011-1050. 22419778 PMC3365860

[R63] Wayman GA, Impey S, Marks D, Saneyoshi T, Grant WF, Derkach V, Soderling TR, 2006. Activity-dependent dendritic arborization mediated by CaM-kinase I activation and enhanced CREB-dependent transcription of wnt-2. Neuron 50 (6), 897–909. 10.1016/j.neuron.2006.05.008. 16772171

[R64] Wiedenmann B, Franke WW, 1985. Identification and localization of synaptophysin, an integral membrane glycoprotein of Mr 38,000 characteristic of presynaptic vesicles. Cell 41 (3), 1017–1028. 10.1016/s0092-8674(85)80082-9. 3924408

[R65] Yang D, Kania-Korwel I, Ghogha A, Chen H, Stamou M, Bose DD, Pessah IN, Lehmler HJ, Lein PJ, 2014. PCB 136 atropselectively alters morphometric and functional parameters of neuronal connectivity in cultured rat hippocampal neurons via ryanodine receptor-dependent mechanisms. Toxicol. Sci 138 (2), 379–392. 10.1093/toxsci/kft334. 24385416 PMC4007107

[R66] Zimmer B, Angioni C, Osthues T, Toewe A, Thomas D, Pierre SC, Geisslinger G, Scholich K, Sisignano M, 2018. The oxidized linoleic acid metabolite 12,13-DiHOME mediates thermal hyperalgesia during inflammatory pain. Biochim. Biophys. Acta, Mol. Cell Biol. Lipids 1863 (7), 669–678. 10.1016/j.bbalip.2018.03.012. 29625231

[R67] Zoeller RT, Vandenberg LN, 2015. Assessing dose-response relationships for endocrine disrupting chemicals (EDCs): a focus on non-monotonicity. Environ. Health: A Global Access Science Source 14 (1), 1–5. 10.1186/s12940-015-0029-4. PMC4440251 25971795

[R68] Zou R, El Marroun H, Voortman T, Hillegers M, White T, Tiemeier H, 2021. Maternal polyunsaturated fatty acids during pregnancy and offspring brain development in childhood. Am. J. Clin. Nutr 114 (1), 124–133. 10.1093/ajcn/nqab049. 33742211

