# Supplementary material for: Oxidized linoleic acid metabolites regulate neuronal morphogenesis in vitro
Source: Neurochem Int. Author manuscript; Available in PMC 2023 Sep 12. (PMC10495953; doi:10.1016/j.neuint.2023.105506)
Supplement: supplemental file 1 [file NIHMS1929247-supplement-supplemental_file_1.docx]

**Supplemental File 1**

Oxidized linoleic acid metabolites regulate neuronal morphogenesis in vitro

Running title: OXLAMs regulate neuronal morphogenesis

Felipe da Costa Souza ^1,2^, Ana Cristina G. Grodzki ^2^, Rhianna M. Morgan ^2^, Zhichao Zhang ^1^, Ameer Y. Taha ^1^ and Pamela J. Lein ^2^ *

Authors and affiliations:

^1^Department of Food Science and Technology, College of Agriculture and Environmental Sciences,

University of California, Davis, California, USA

^2^Department of Molecular Biosciences, School of Veterinary Medicine, University of California, Davis,

California, USA

*Corresponding Author

**
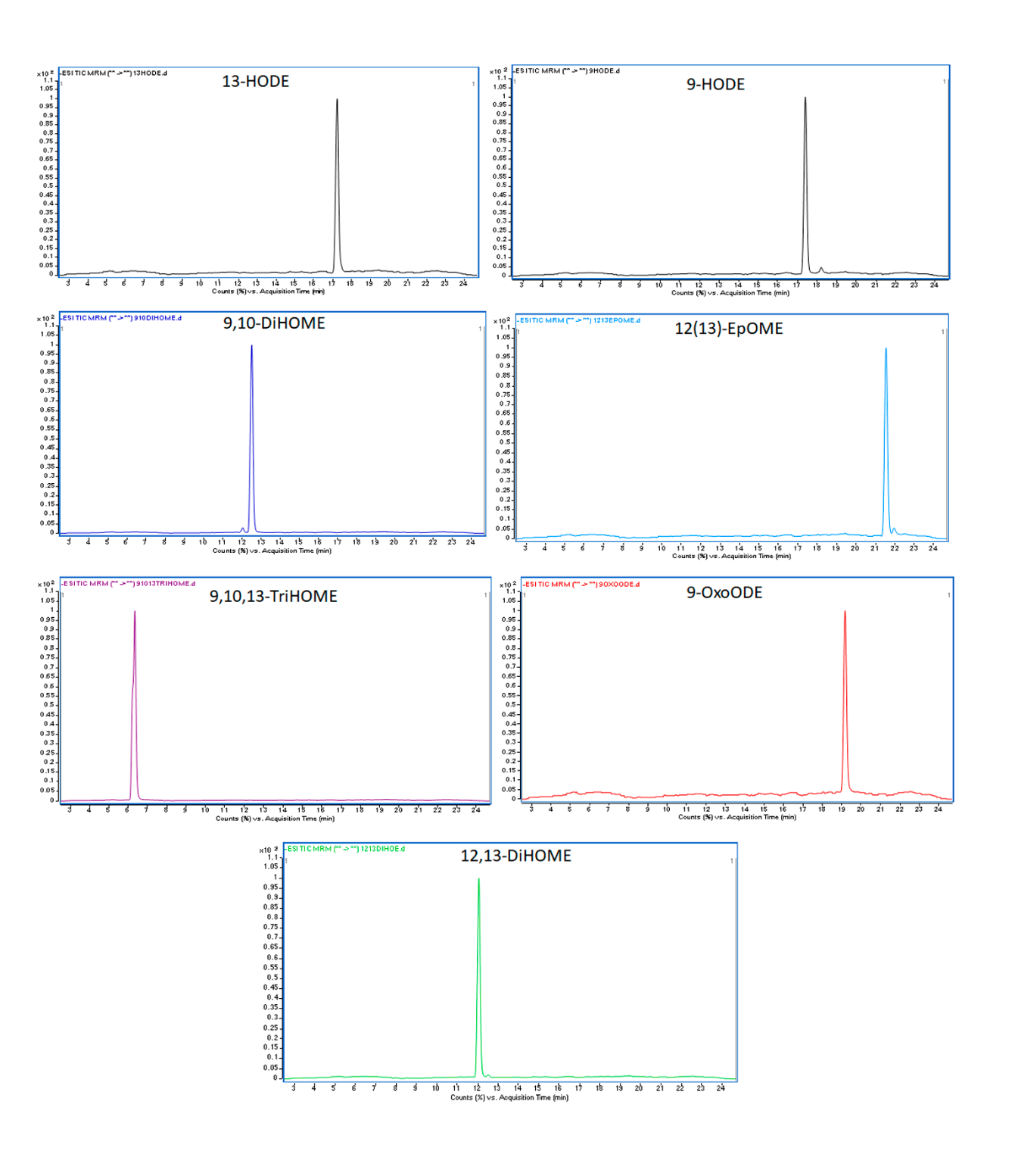
**

Fig S1. Chromatograms showing the purity of 13- HODE; 9-HODE; 9,10-DiHOME; 12(13)-EpOME ; 9,10,13-TriHOME; 9-OxoODE; 12,13-DiHOME stock solutions used for the experiments.


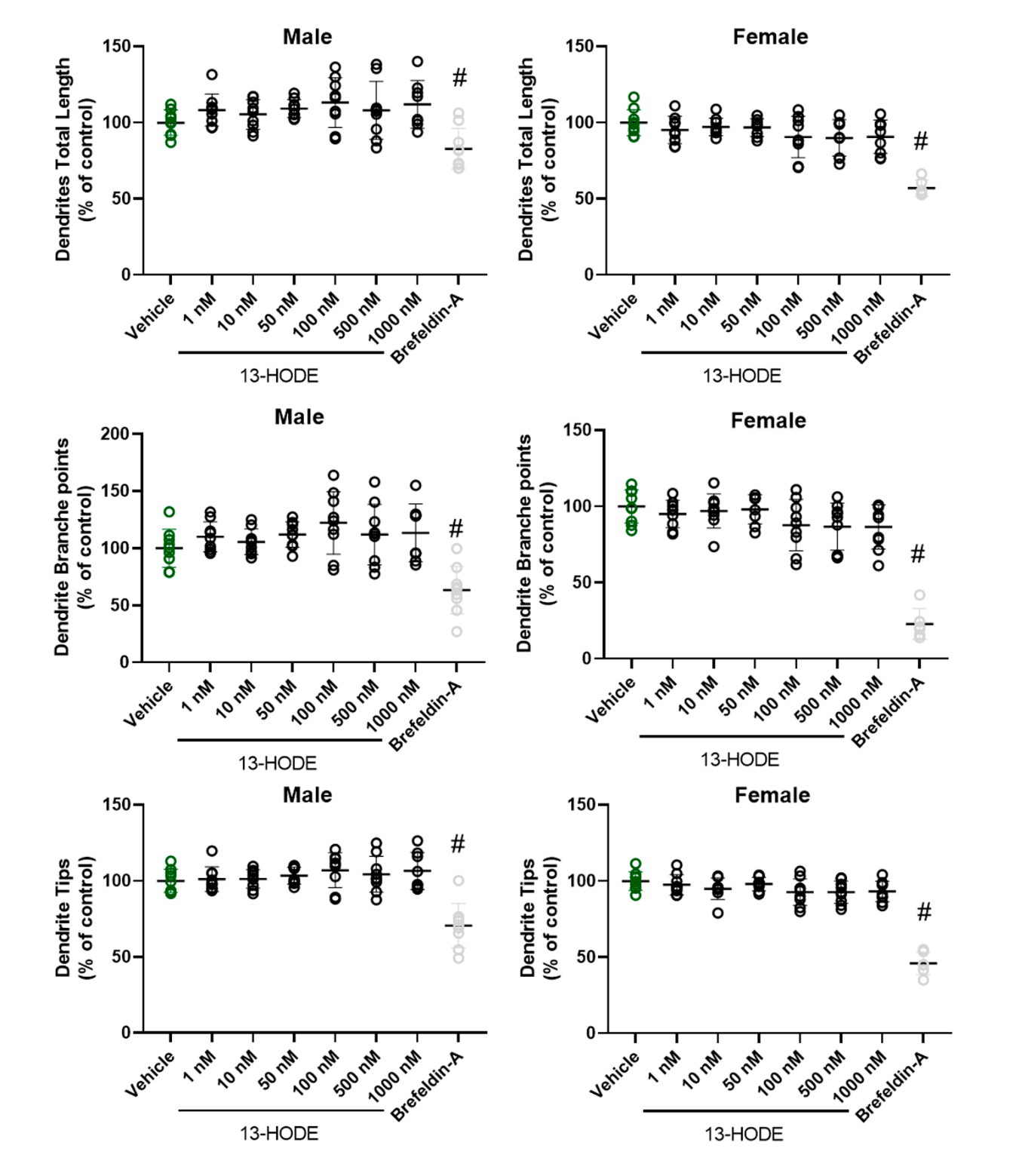


Fig S2. Dendrite total lengh, number of branche points and number of tips of primary rat cortical neurons incubated with different concentrations of 13- HODE. Each figure shows scatter plots with data represented as mean ± SD (n=8-9 wells per treatment per sex from three independent dissections). One-way ANOVA followed by Dunnett’s multiple comparison post hoc test was used to determine concentration-specific effects. Asterisk (*) denotes significance at p < 0.05. Hashtag (#) denotes significance for Brefeldin-A technical control by unpaired t test of sudden p < 0.05.


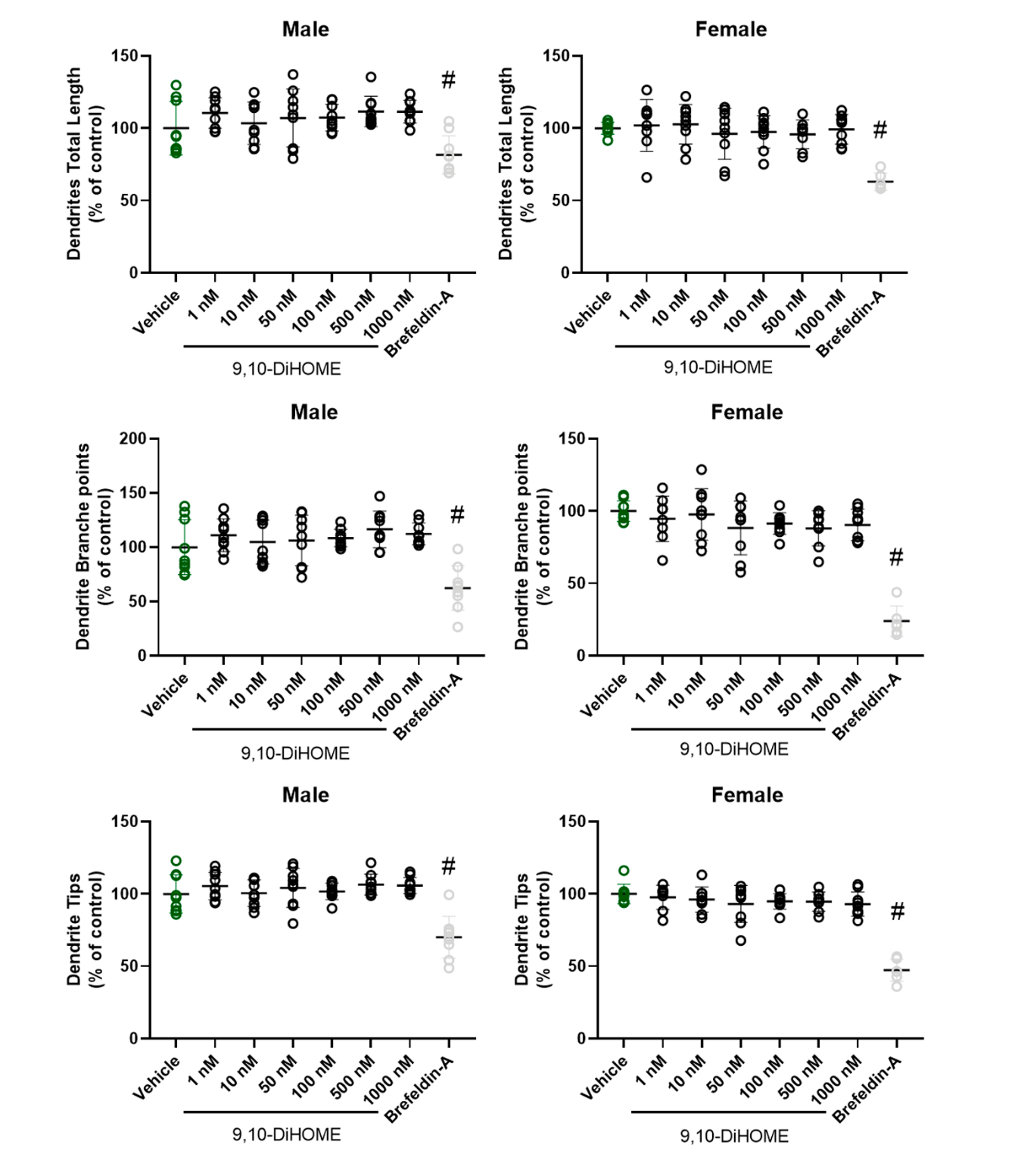


Fig S3. Dendrite total lengh, number of branch points and number of tips of primary rat cortical neurons incubated with different concentrations of 9,10-DiHOME. Each figure shows scatter plots with data represented as mean ± SD (n=8-9 wells per treatment per sex from three independent dissections). One-way ANOVA followed by Dunnett’s multiple comparison post hoc test was used to determine concentration-specific effects. Asterisk (*) denotes significance at p < 0.05. Hashtag (#) denotes significance for Brefeldin-A techical control by unpaired t test of sudden p < 0.05.


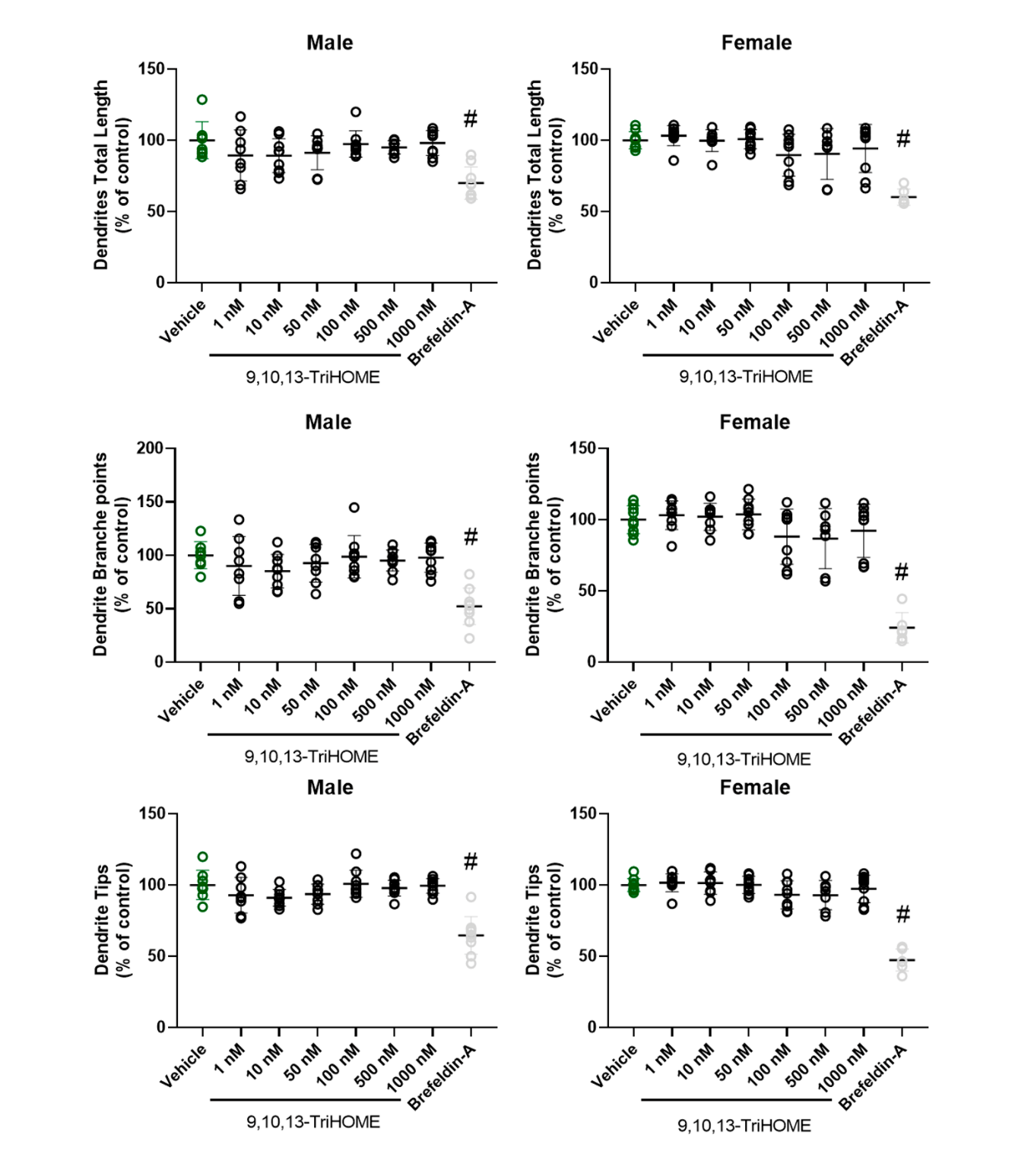


Fig S4. Dendrite total lengh, number of branch points and number of tips of primary rat cortical neurons incubated with different concentrations of 9,10,13-TriHOME. Each figure shows scatter plots with data represented as mean ± SD (n=8-9 wells per treatment per sex from three independent dissections). One-way ANOVA followed by Dunnett’s multiple comparison post hoc test was used to determine concentration-specific effects. Asterisk (*) denotes significance at p < 0.05. Hashtag (#) denotes significance for Brefeldin-A techical control by unpaired t test of sudden p < 0.05.


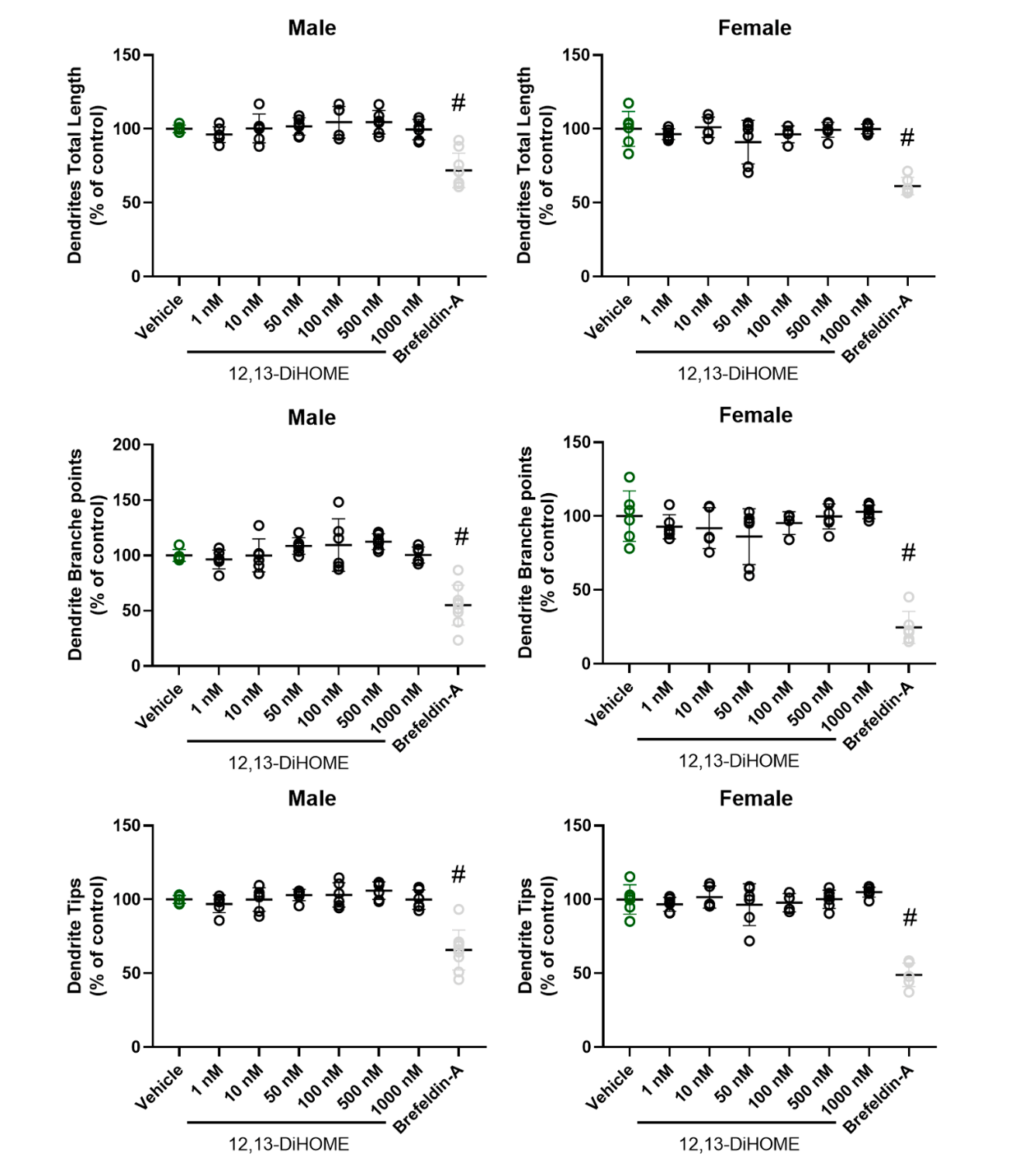


Fig S5. Dendrite total lengh, number of branch points and number of tips of primary rat cortical neurons incubated with different concentrations of 12,13-DiHOME. Each figure shows scatter plots with data represented as mean ± SD (n=8-9 wells per treatment per sex from three independent dissections). One-way ANOVA followed by Dunnett’s multiple comparison post hoc test was used to determine concentration-specific effects. Asterisk (*) denotes significance at p < 0.05. Hashtag (#) denotes significance for Brefeldin-A techical control by unpaired t test of sudden p < 0.05.


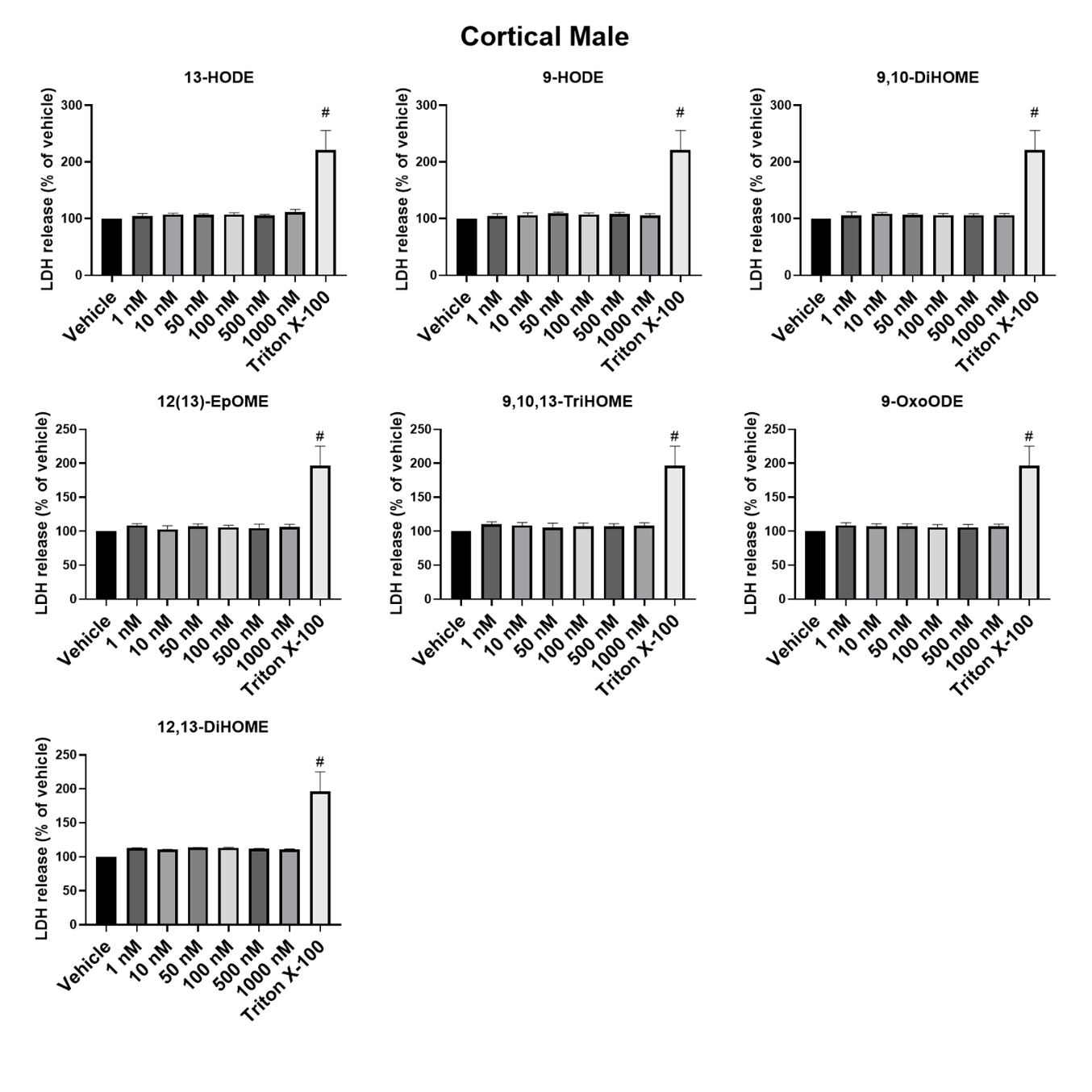


Fig S6. Lactate dehydrogenase (LDH) release in primary rat male cortical neurons measured, from cell supernatant after 48 h of treatment with different concentrations of 13- HODE; 9-HODE; 9,10-DiHOME; 12(13)-EpOME; 9,10,13-TriHOME; 9-OxoODE; and 12,13-DiHOME. Triton X-100 served as a lysed cell control for viability. Each figure shows data represented as mean ± SD (n=3 from three independent dissections). One-way ANOVA followed by Dunnett’s multiple comparison post hoc test was used to determine concentration-specific effects. No significant effect was observed. Triton X-100, a technical positive control, resulted in reduced viability and increased LDH release.


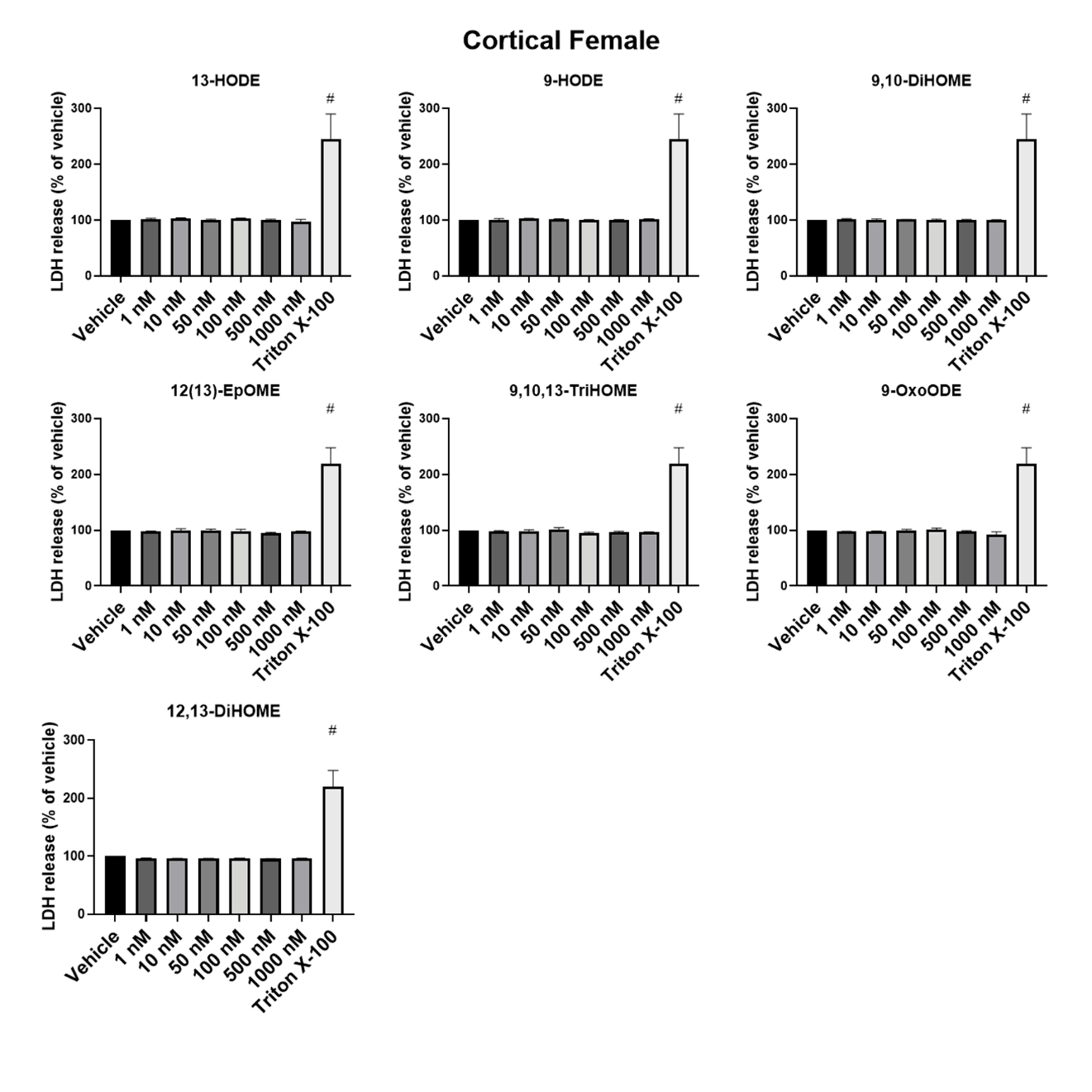


Fig S7. Lactate dehydrogenase (LDH) release in primary rat female cortical neurons measured from cell supernatant after 48 h of treatment with different concentrations of 13-HODE; 9-HODE; 9,10-DiHOME; 12(13)-EpOME; 9,10,13-TriHOME; 9-OxoODE; and 12,13-DiHOME. Triton X-100 served as a lysed cell control for viability Each figure shows data represented as mean ± SD (n=3 from three independent dissections). One-way ANOVA followed by Dunnett’s multiple comparison post hoc test was used to determine concentration-specific effects. No significant effect was observed.


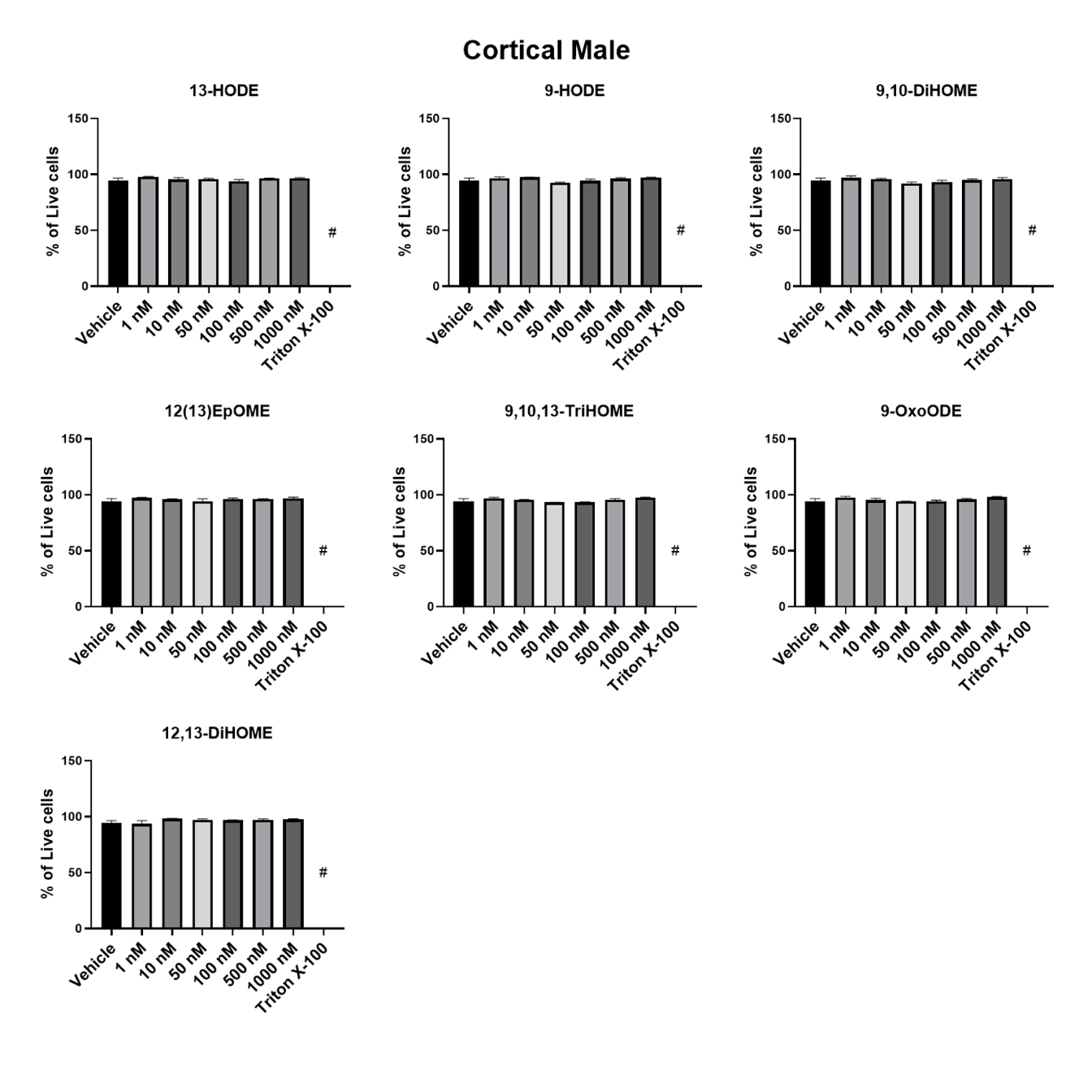


Fig S8. Cell viability on primary rat male cortical neurons measured after 48 h of treatment with different concentrations of 13-HODE; 9-HODE; 9,10-DiHOME; 12(13)-EpOME; 9,10,13-TriHOME; 9-OxoODE; and 12,13-DiHOME. Cells were incubated with calcein-AM and Hoechst-33342. Graphics are showing % of live cells (calcein-AM positive) from total nuclei (Hoechst-33342 stained) ± SD. Triton X-100 served as a lysed cell control for viability (n=3 from three independent dissections). One-way ANOVA followed by Dunnett’s multiple comparison post hoc test was used to determine concentration-specific effects. No significant effect was observed.


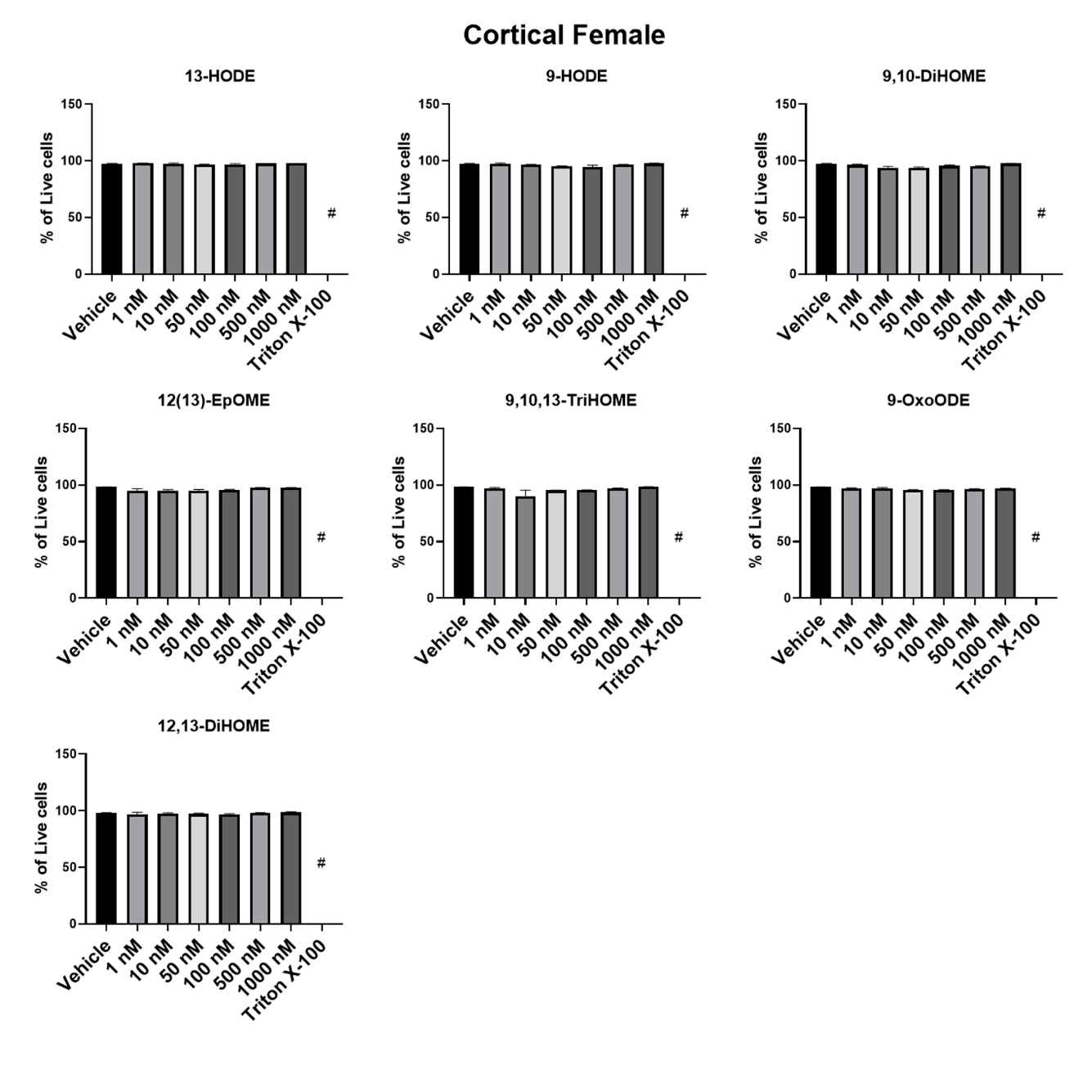


Fig S9. Cell viability on primary rat female cortical neurons measured after 48 h of treatment with different concentrations of 13-HODE; 9-HODE; 9,10-DiHOME; 12(13)-EpOME; 9,10,13-TriHOME; 9-OxoODE; and 12,13-DiHOME. Cells were incubated with calcein-AM and Hoechst-33342. Graphics are showing % of live cells (calcein-AM positives) from total nuclei (Hoechst-33342 stained) ± SD. Triton X-100 served as a lysed cell control for viability. (n=3 from three independent dissections). One-way ANOVA followed by Dunnett’s multiple comparison post hoc test was used to determine concentration-specific effects. No significant effect was observed.


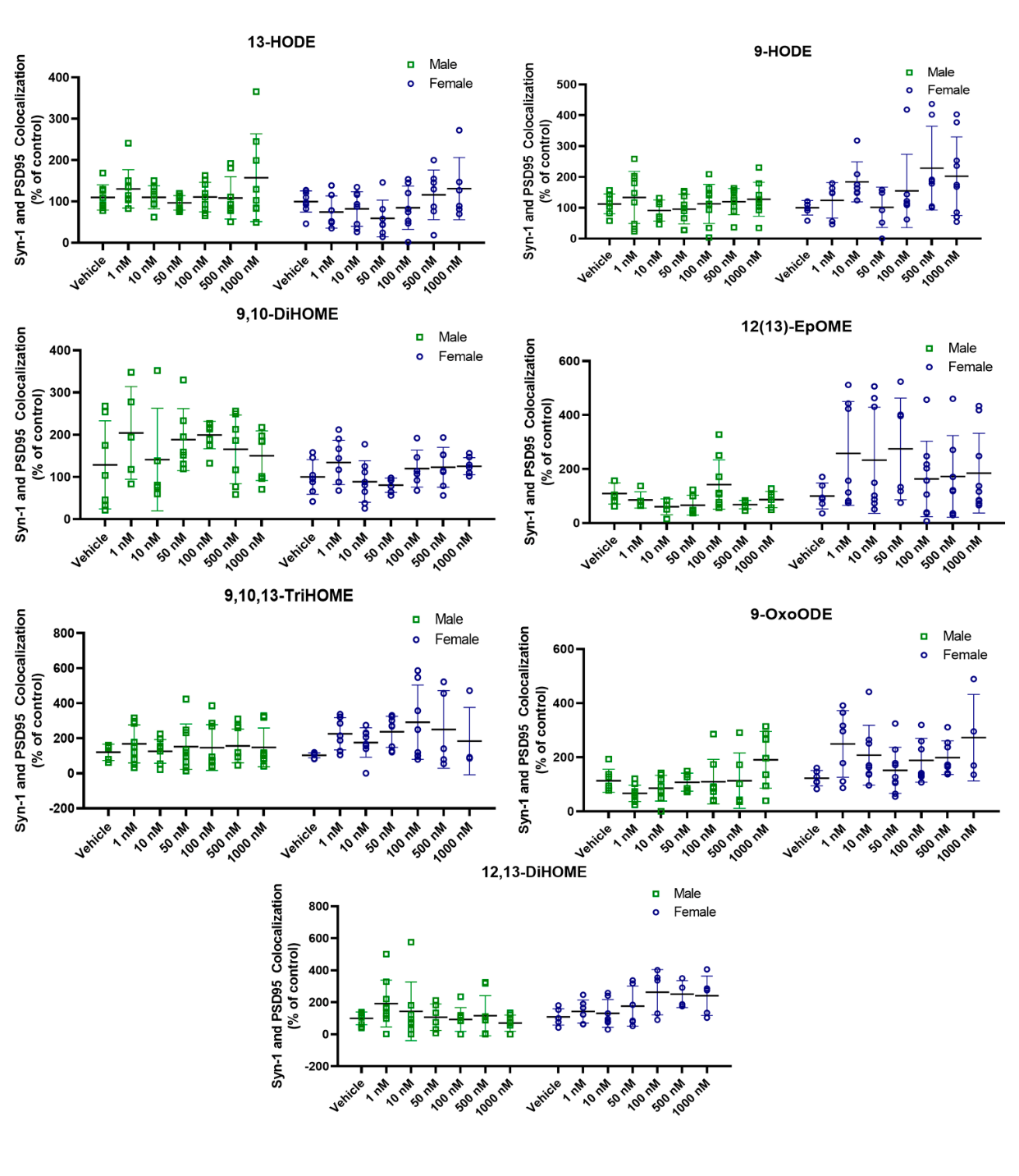


Fig S10. Synaptic connectivity on primary rat male and female cortical neurons measured after 48 h of treatment with different concentrations of 13- HODE; 9-HODE; 9,10-DiHOME; 12(13)-EpOME; 9,10,13-TriHOME; 9-OxoODE; and 12,13-DiHOME. After treatment, cells fixed and immunostained for synaptophysin 1, PSD95 and MAP2B . Graphics are showing synaptophysin 1 and PSD95 colocalization along MAP2B, as % of vehicle ± SD (n=9-12 wells per treatment per sex from four independent dissections). One-way ANOVA followed by Dunnett’s multiple comparison post hoc test was used to determine concentration-specific effects. No significant effect was observed.
