# Supplementary material for: Oxidized linoleic acid metabolites regulate neuronal morphogenesis in vitro
Source: Neurochem Int. Author manuscript; Available in PMC 2023 Sep 12. (PMC10495953; doi:10.1016/j.neuint.2023.105506)
Supplement: supplemental file 2 [file NIHMS1929247-supplement-supplemental_file_2.docx]

**Supplemental File 2**

**Automated high content imaging and analysis**

Oxidized linoleic acid metabolites regulate neuronal morphogenesis in vitro

Running title: OXLAMs regulate neuronal morphogenesis

Felipe da Costa Souza ^1,2^, Ana Cristina G. Grodzki ^2^, Rhianna M. Morgan ^2^, Zhichao Zhang ^1^, Ameer Y. Taha ^1^ and Pamela J. Lein ^2^ *

Authors and affiliations:

^1^Department of Food Science and Technology, College of Agriculture and Environmental Sciences,

University of California, Davis, California, USA

^2^Department of Molecular Biosciences, School of Veterinary Medicine, University of California, Davis,

California, USA

*Corresponding Author

All culture experiments in 24-well plates (axon outgrowth, dendritic arborization and synaptic connectivity) were conducted with cells plated and later imaged on 12mm round glass coverslips (BellCo; Cat #1943-10012A).

For axon outgrowth and dendritic arborization experiments, 25 sites per coverslip (as 5x5 adjacent sites) were captured using a 10x objective, with three coverslips per experimental group, per dissection, from three independent dissections. Dendritic arborization was analyzed using the automated unbiased extension of Omnisphero software as described by Schmuck et al., 2020 (Schmuck et al., 2020). Axon outgrowth was analyzed using a cell scoring custom built journal in MetaXpress Custom Module Editor, MetaXpress software (Molecular Devices; version 5.3.0.5). The journal starts with the “Setup” (Step 1, Fig S11), addressing names to each captured channel. Next, the “Count Nuclei Objects” using the corresponding channel for DAPI staining built a mask considering round objects with minimum width of 8 µm and maximum of 28 µm (Step 2, Fig S11). To find neurites, the next algorithm uses the corresponding channel for TAU1 staining separates cell bodies and neurites by size and width, using a maximum width of 7 µm for neurites and 25 µm for cell bodies. Nuclei staining is also used to colocalize with the predicted cell bodies (Step 3, Fig S11). The final step measures the mask built for neurites and normalize it with number of nuclei objects (Step 4, Fig S11). The intensity above background used for each algorithm to draw the masks was determined for each staining and within each individual experiment, and the determined number was kept same to all experimental groups.


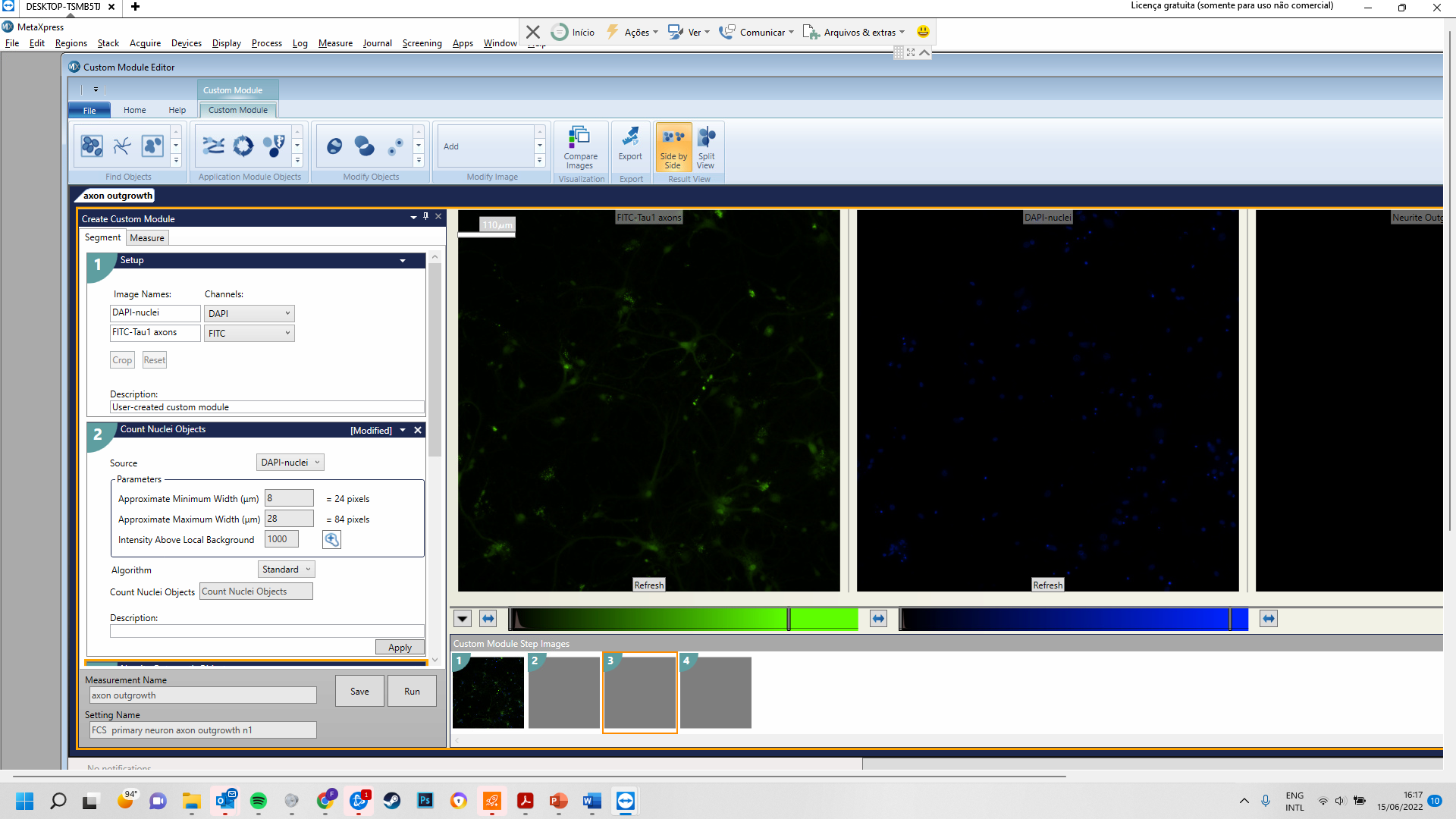

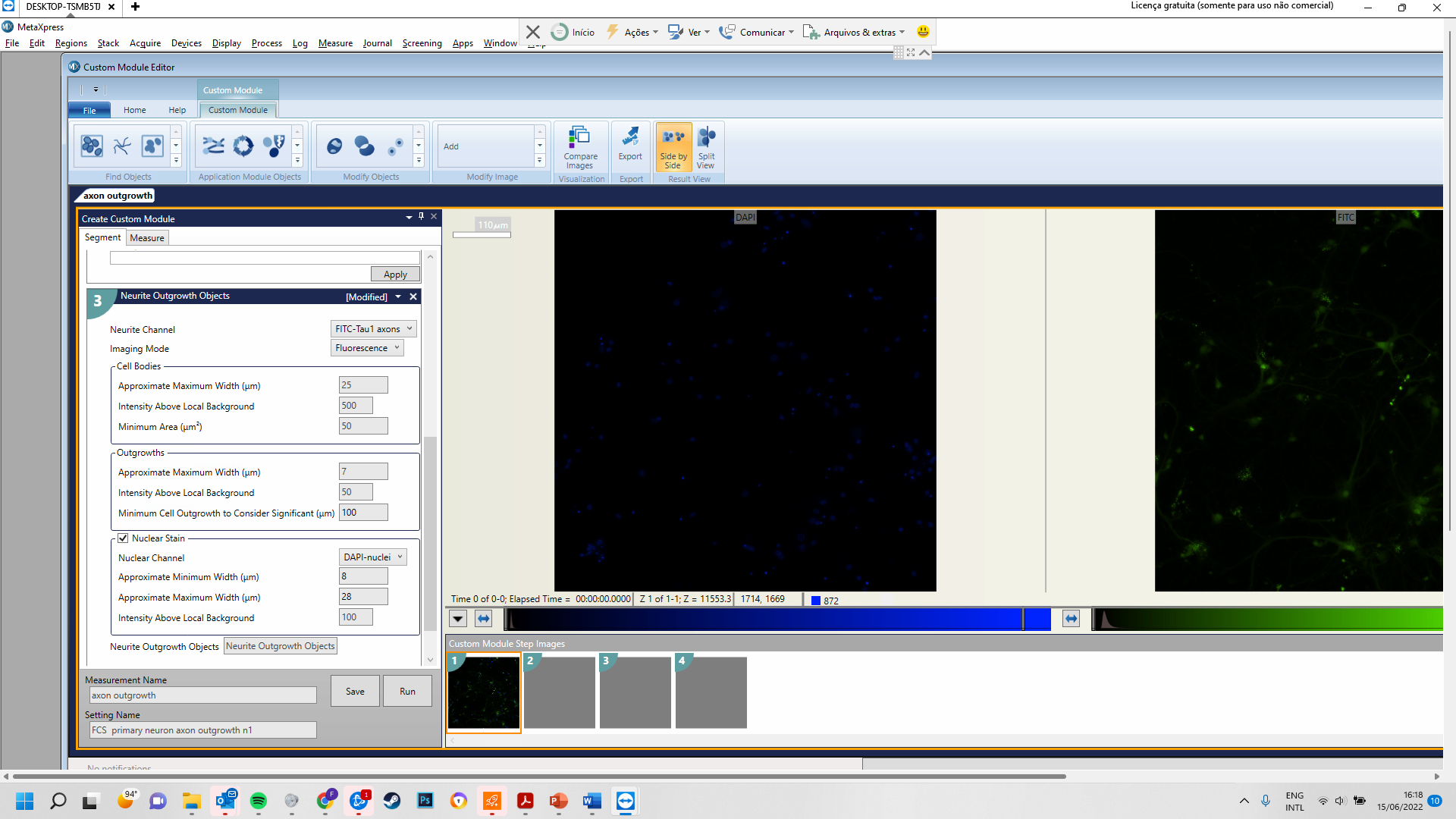

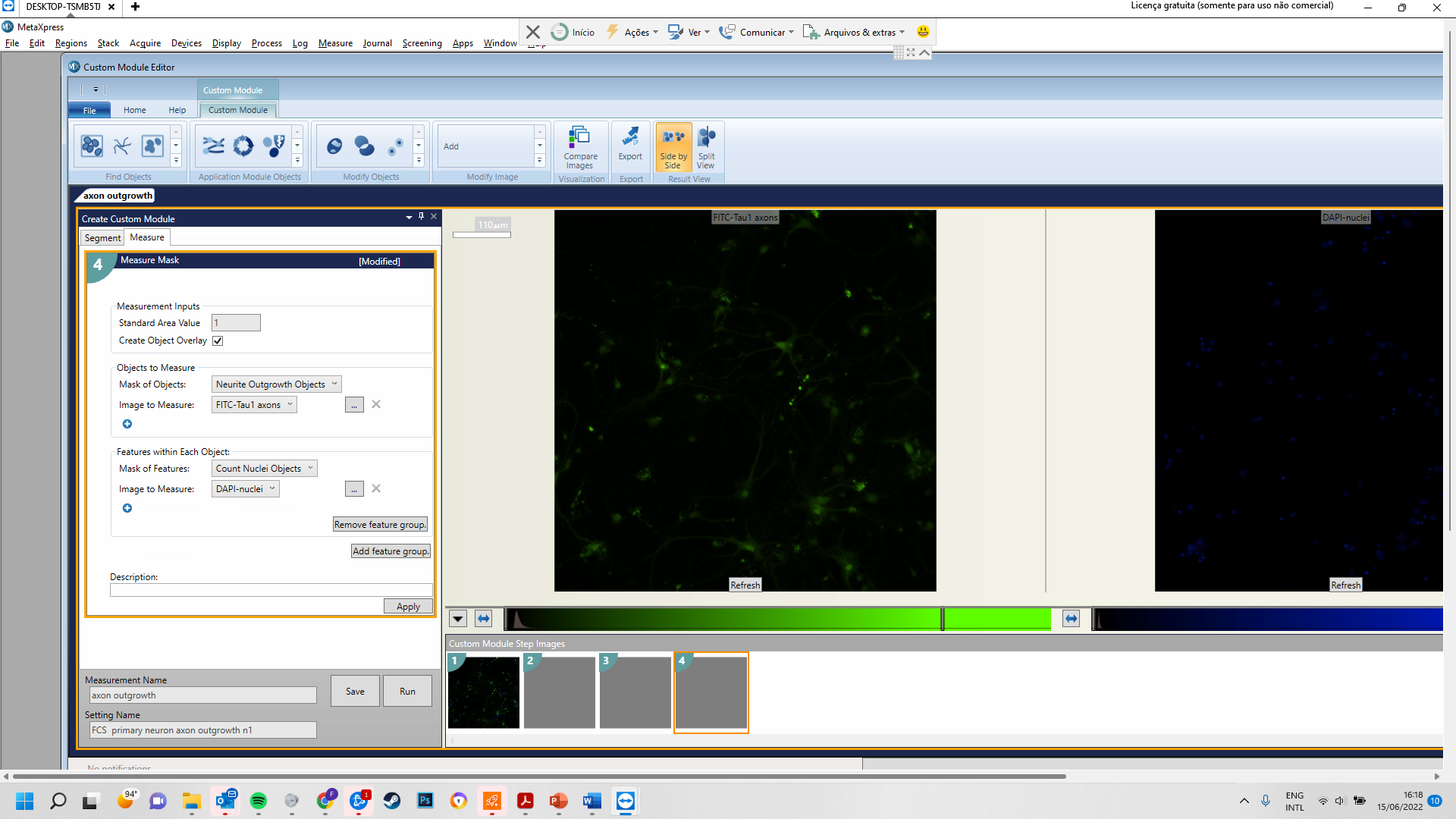

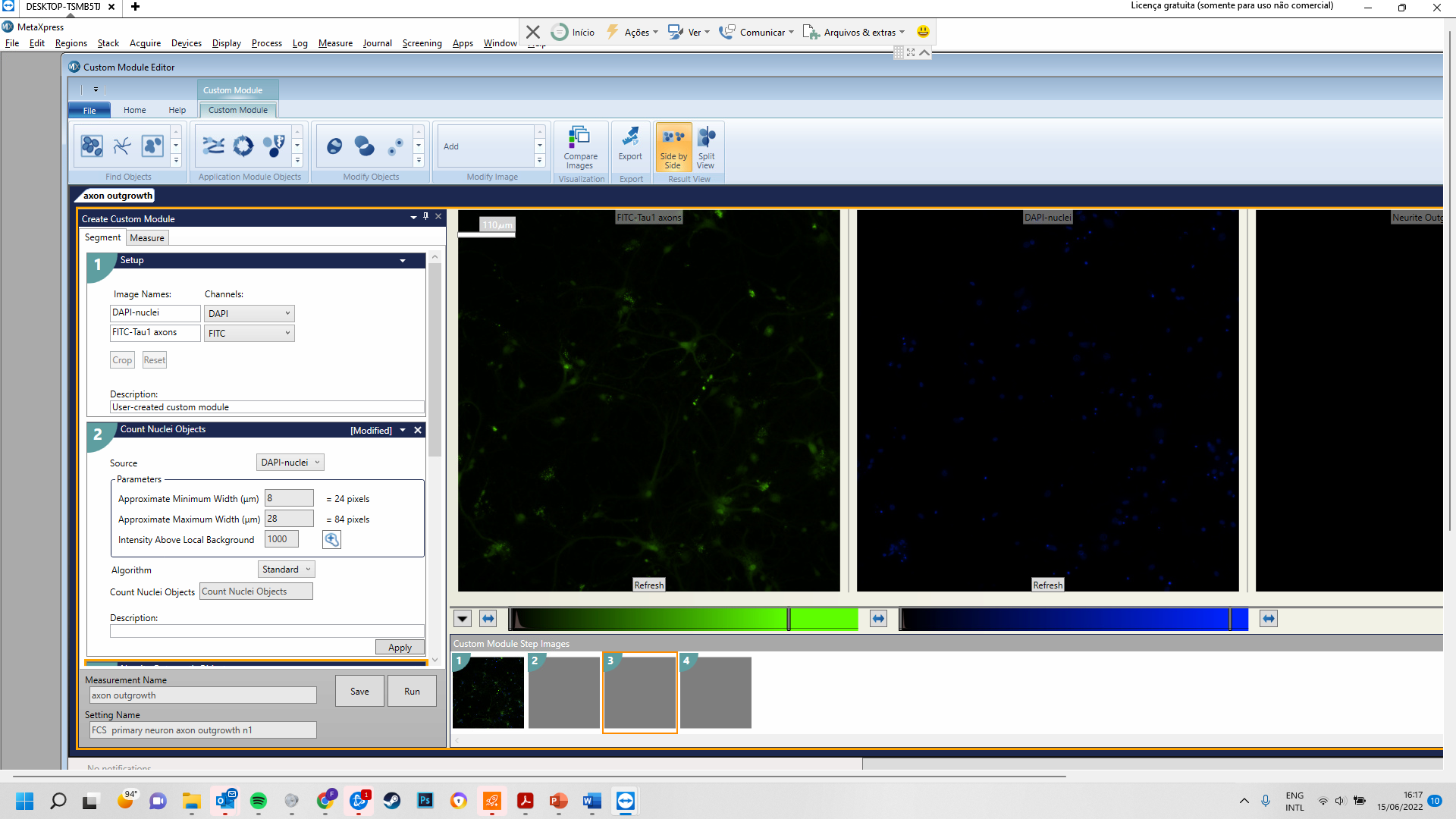


Fig S11. Example of the axon outgrowth analysis custom built using MetaXpress Custom Module Editor (version 5.3.0.5).

The calcein AM staining was conducted in 96-well plates, with 5 sites per well (spaced 500 µm in each direction) captured using a 20x objective, with 5-6 wells per experimental group, per dissection, from three independent dissections. To quantify calcein-AM-positive (live) cells, a cell scoring journal was built in MetaXpress software (Molecular Devices; version 5.3.0.5), using the software MetaXpress Custom Module Editor. The journal starts with the “Setup” (Step 1, Fig S13), addressing names to each wavelength captured (channel). Next, the “Find Round Objects” step using the corresponding channel for DAPI staining built a nuclei mask considering round objects with minimum width of 8 µm and maximum of 28 µm (Step 2, Fig S13). Next, all edge nuclei were excluded from the mask with “Remove Border Objects” (Step 3, Fig S13) to reduce artifact. An “Adaptive Threshold” algorithm (Step 4, Fig S13) built masks for calcein AM staining, and a “Logical Operations” step (Step 5, Fig S13) finds colocalized nuclei and calcein AM, generating a “Live cells” mask. The final journal measures the total number of cells from the nuclei mask and total number of live cells from the live cells mask (Step 6, Fig S13). The intensity above background used for each journal to draw the masks was determined for each staining and within each individual experiment, and the determined threshold was kept the same to all samples in the same experimental groups.


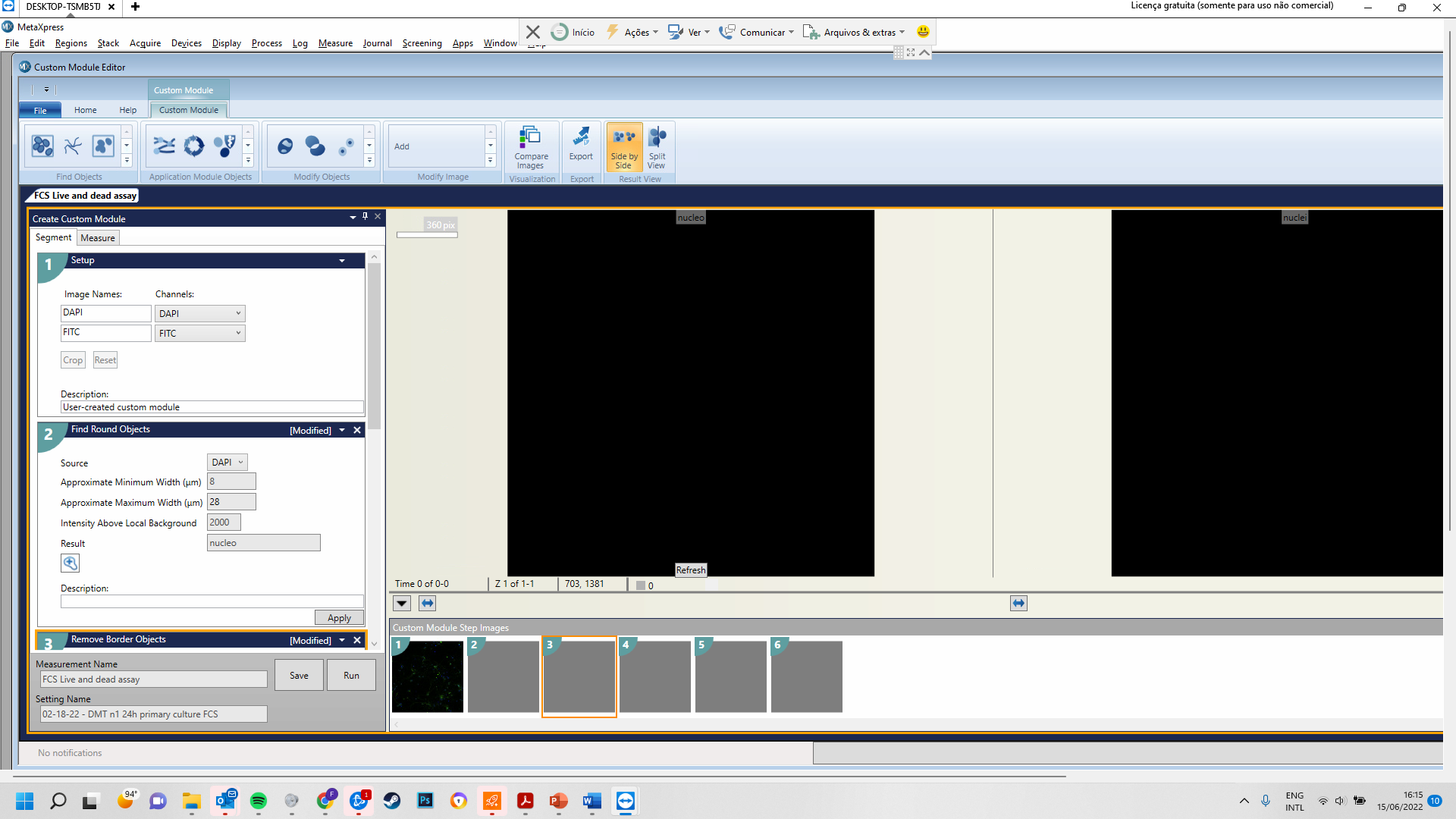

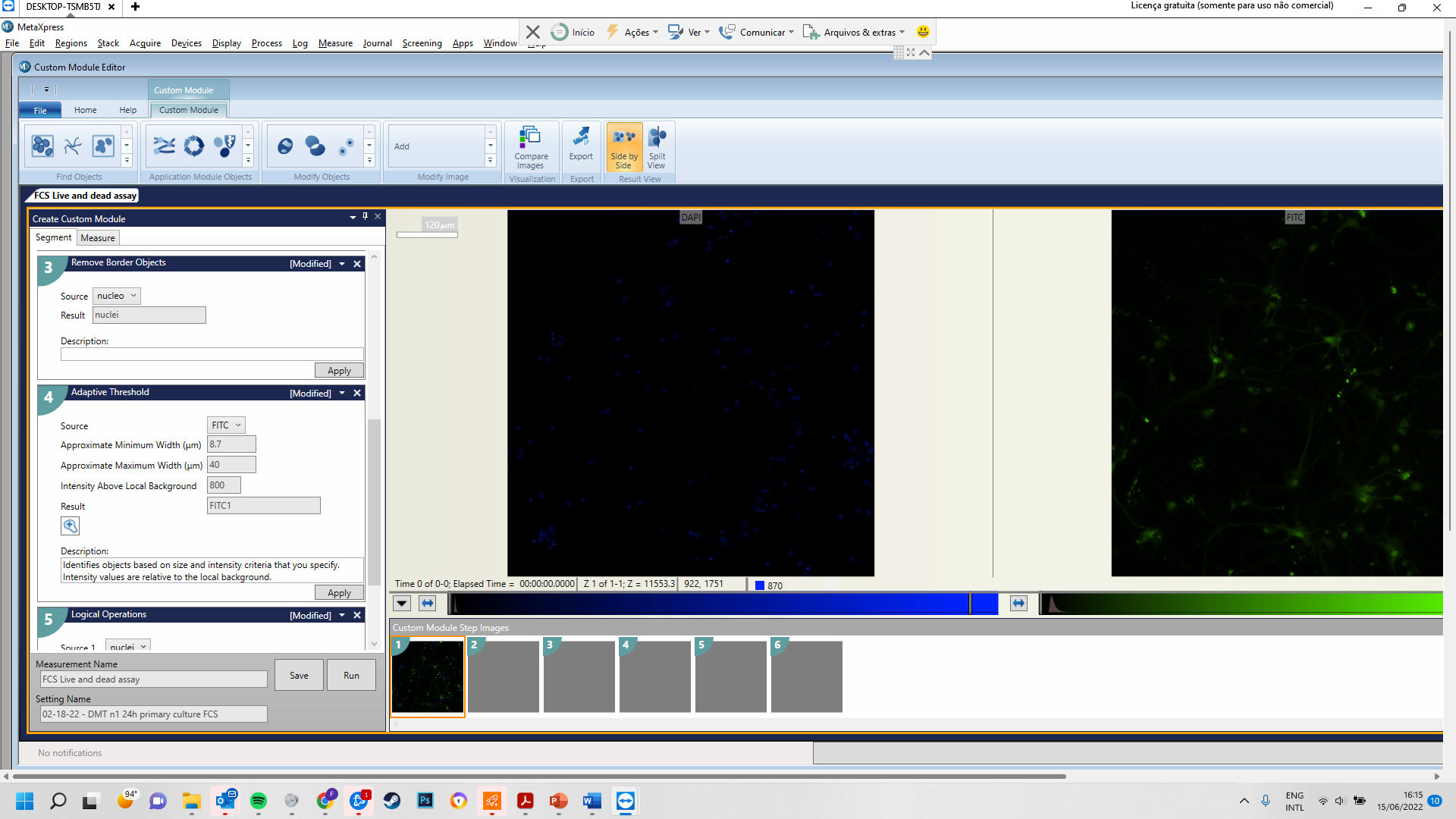

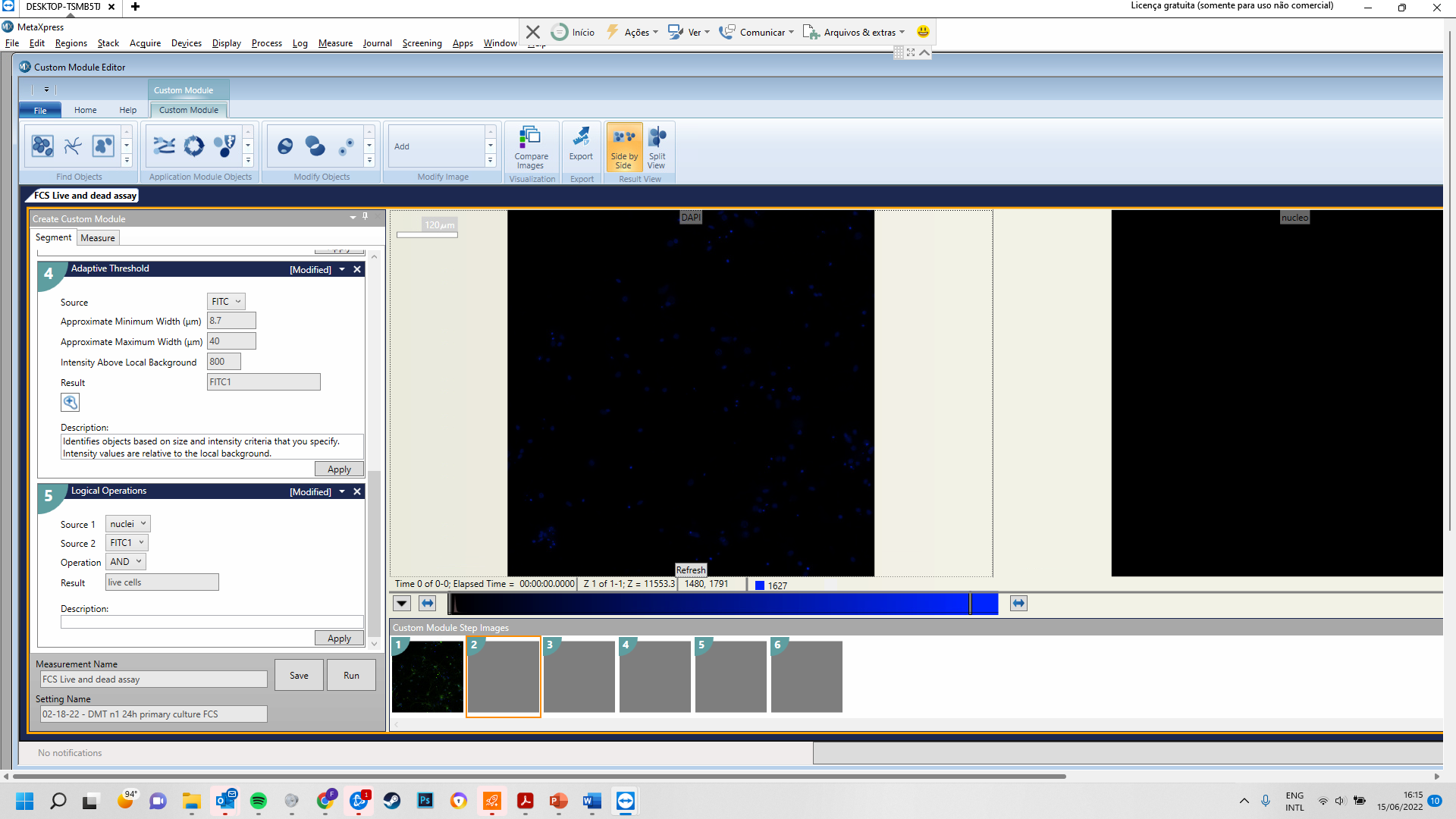

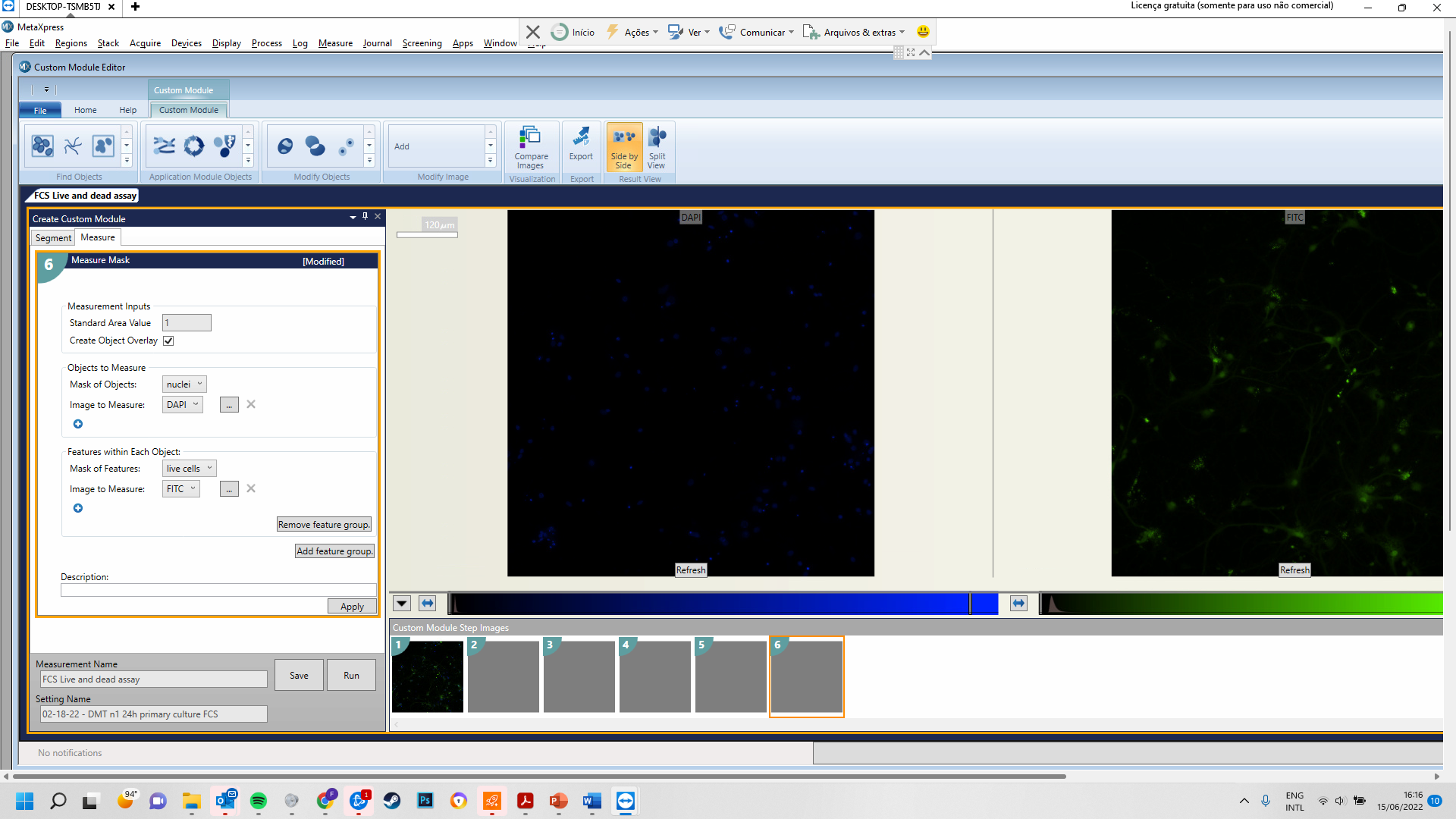

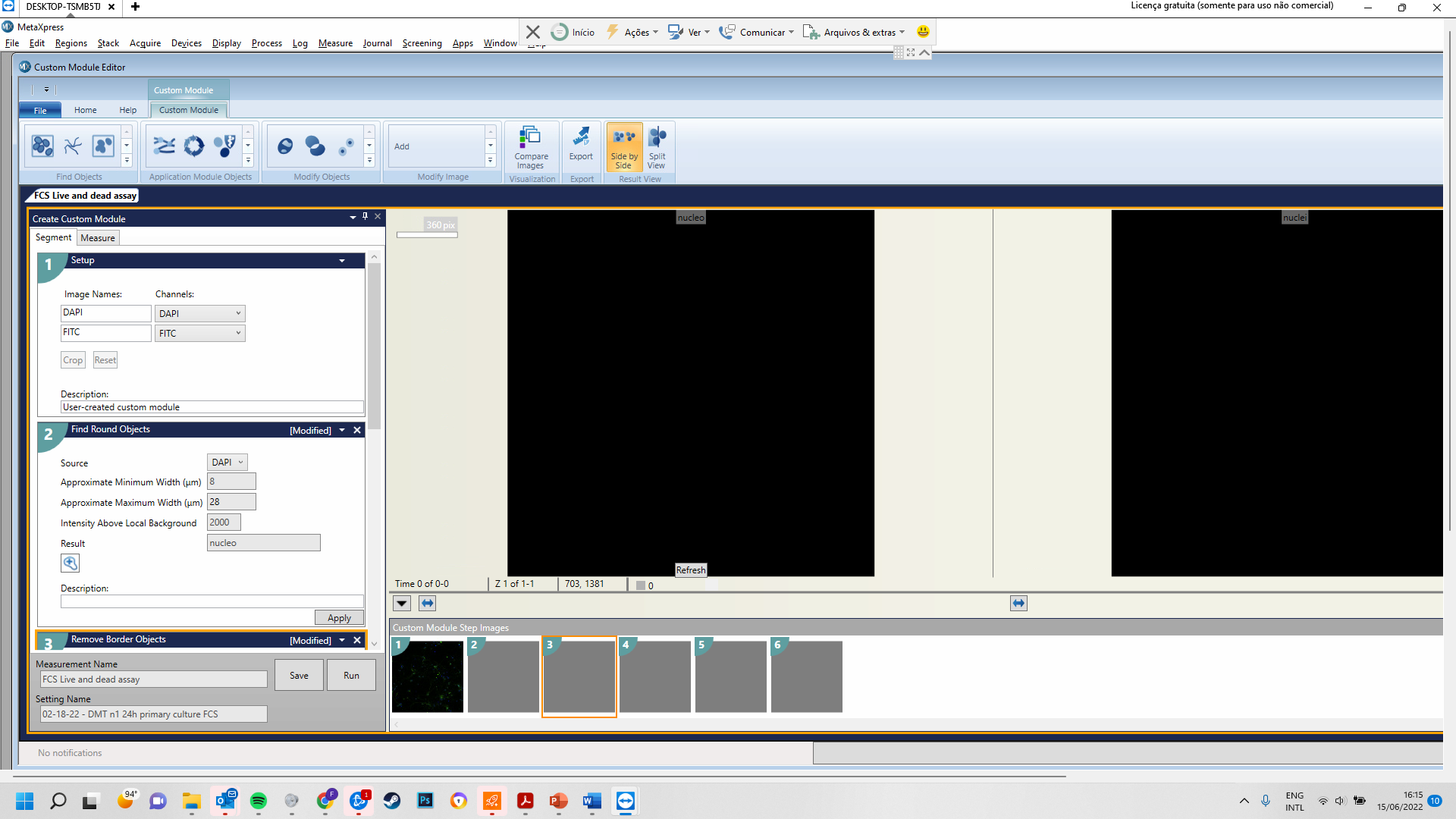

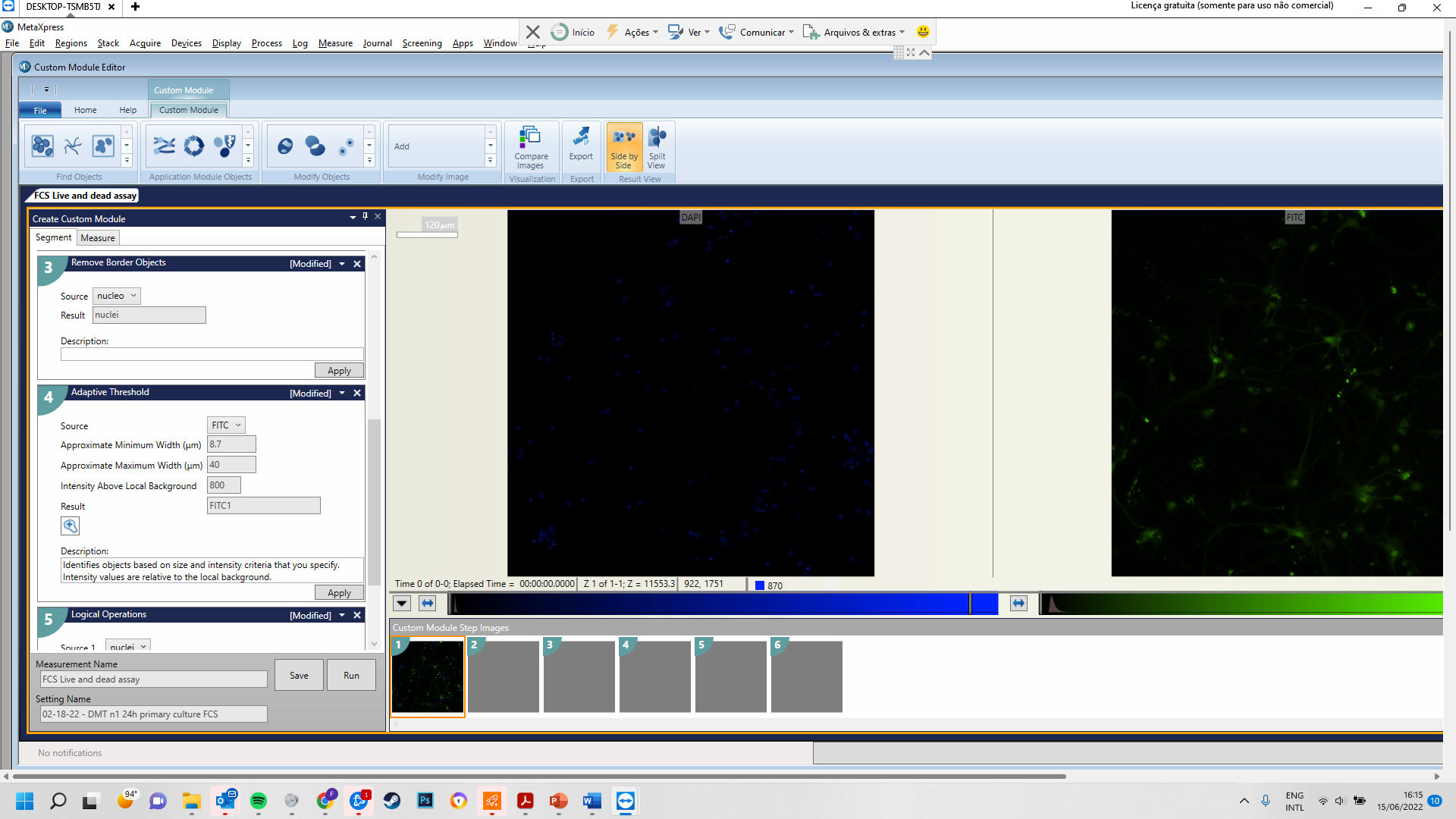


Fig S12. Example of the calcein AM analysis built using MetaXpress Custom Module Editor (version 5.3.0.5).

For synaptic connectivity, 20 sites per coverslip (as 4x5 sites spaced 500 µm in each direction) were captured using a 40x objective, with three coverslips per experimental group, per dissection, from four independent dissections. The area of synaptic puncta along MAP2-positive processes was measured using cell scoring custom built journal in MetaXpress software (Molecular Devices; version 5.3.0.5), using the software MetaXpress Custom Module Editor. The journal starts with the “Setup” step (Step 1, Fig S12), addressing names to each wavelength (channel) captured. Next, the “Adaptive Threshold” step using the corresponding channel for MAP2B staining built a mask on objects with minimum width of 1 µm and maximum of 5 µm (Step 2, Fig S12). The “find round objects” step using the corresponding channel for synaptophysin 1 staining built a mask to identify synaptophysin 1 puncta, using a maximum width of 1 µm (Step 3, Fig S12). A “Logical Operations” step finds colocalized synaptophysin 1 puncta along MAP2B mask, excluding the puncta that are out of the MAP2B mask (Step 4, Fig S12). The process repeats for PSD95 staining, with the “find round objects” and “Logical Operations” journals identifying PSD95 puncta along the MAP2B mask (Step 5, 6, Fig S12). Another “Logical Operations” finds the colocalization of synaptophysin 1 and PSD95 puncta along MAP2B, using the images previously generated by the “Logical Operations” in steps 4 and 6 (Step 7, Fig S12). The final segment (measures to be analyzed and displayed) (Step 8, Fig S12) measures the mask built for MAP2B in step 2, for synaptophysin 1 along MAP2B in step 4, for PSD95 along MAP2B in step 6, and for colocalized synaptophysin 1/PSD95 puncta along MAP2B in step 7.

A completely new analysis with a “Count Nuclei Objects” journal (identical to step 2, Fig S11), counts the number of nuclei using DAPI staining. The number of nuclei was used to normalize synaptophysin 1/PSD95 puncta along MAP2B data with the total number of cells. The intensity above background used for each journal to draw the masks was determined for each staining and within each individual experiment, and the determined threshold was kept the same for all the samples in the same experimental groups.


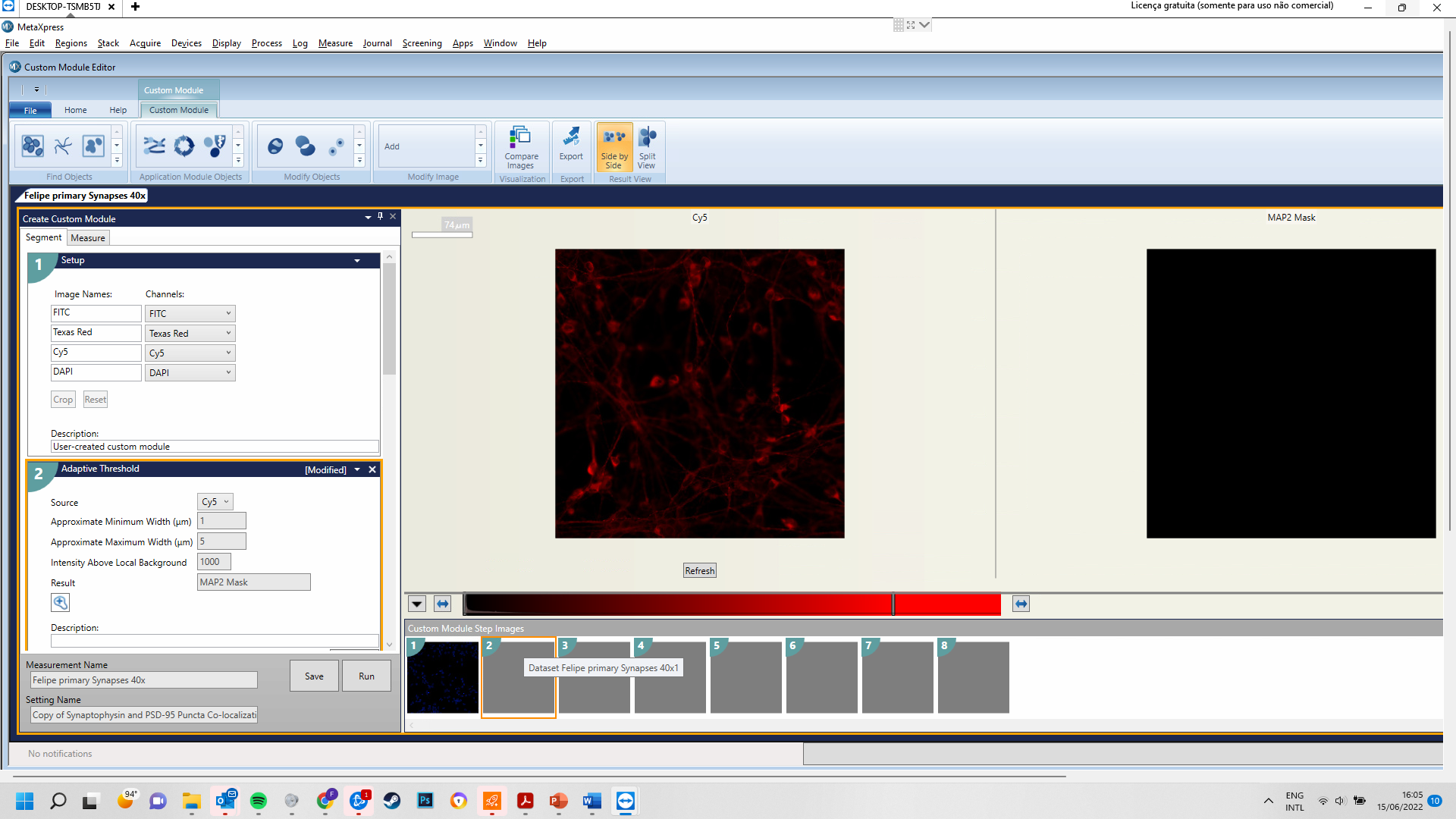

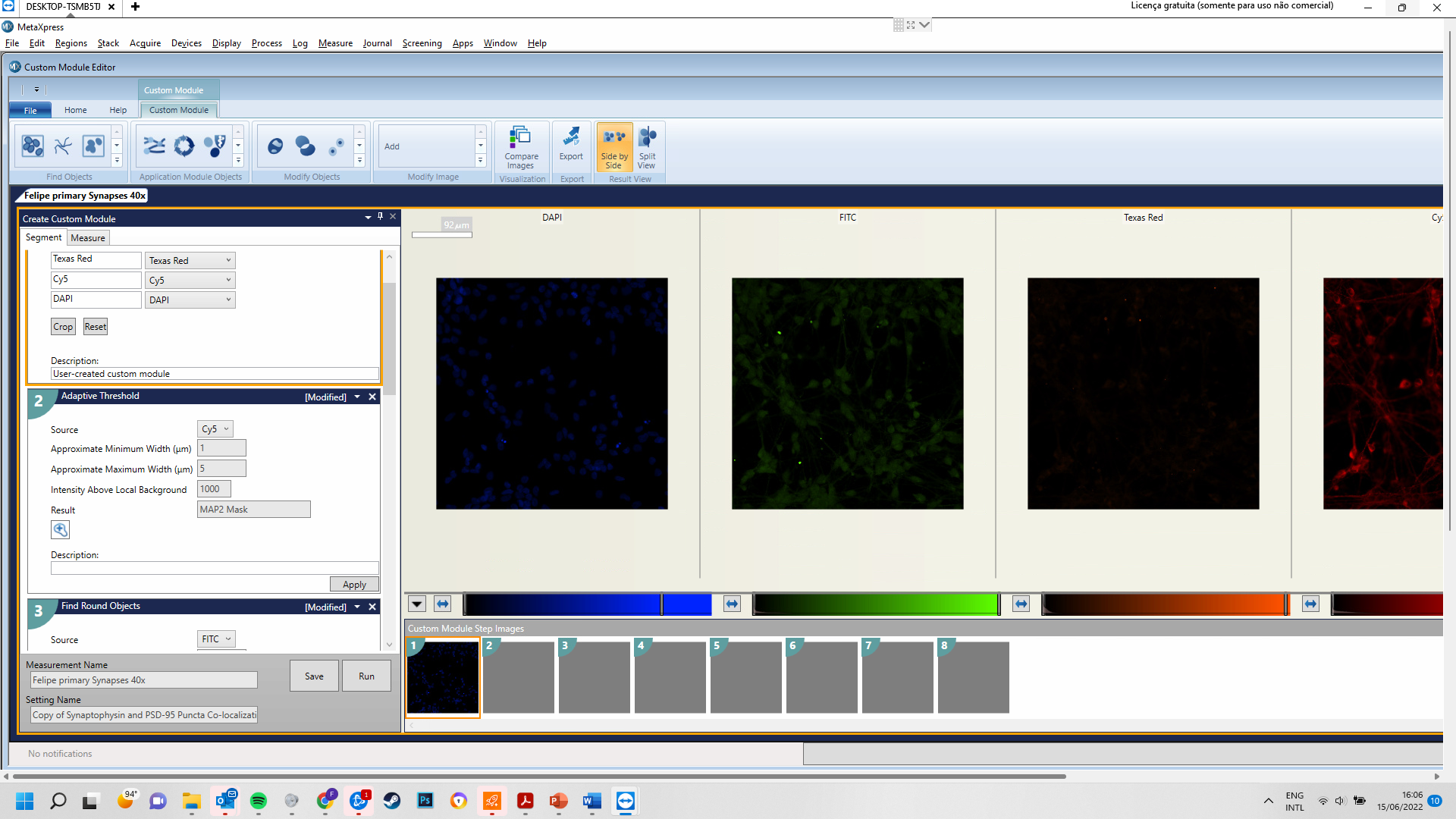

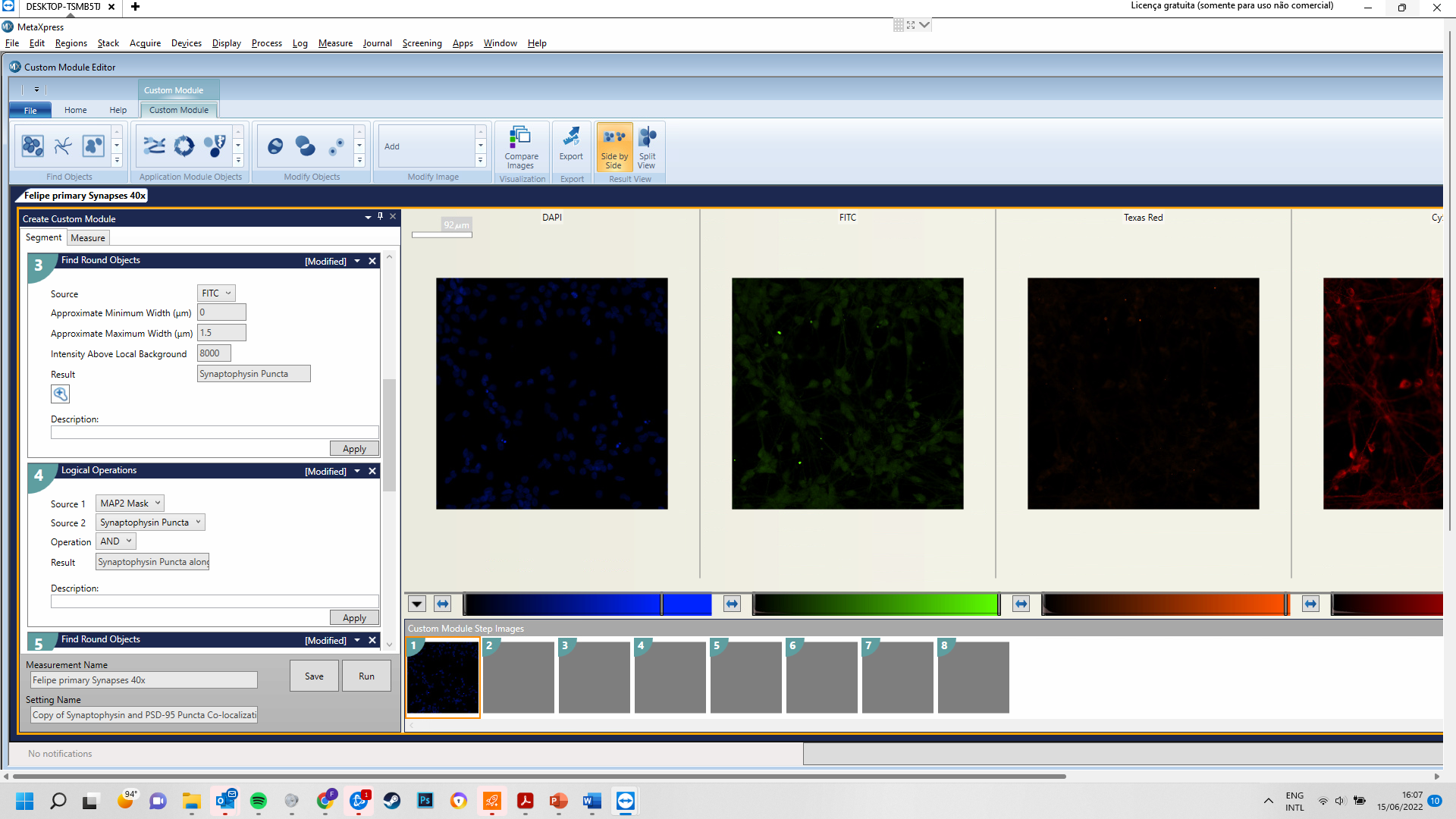

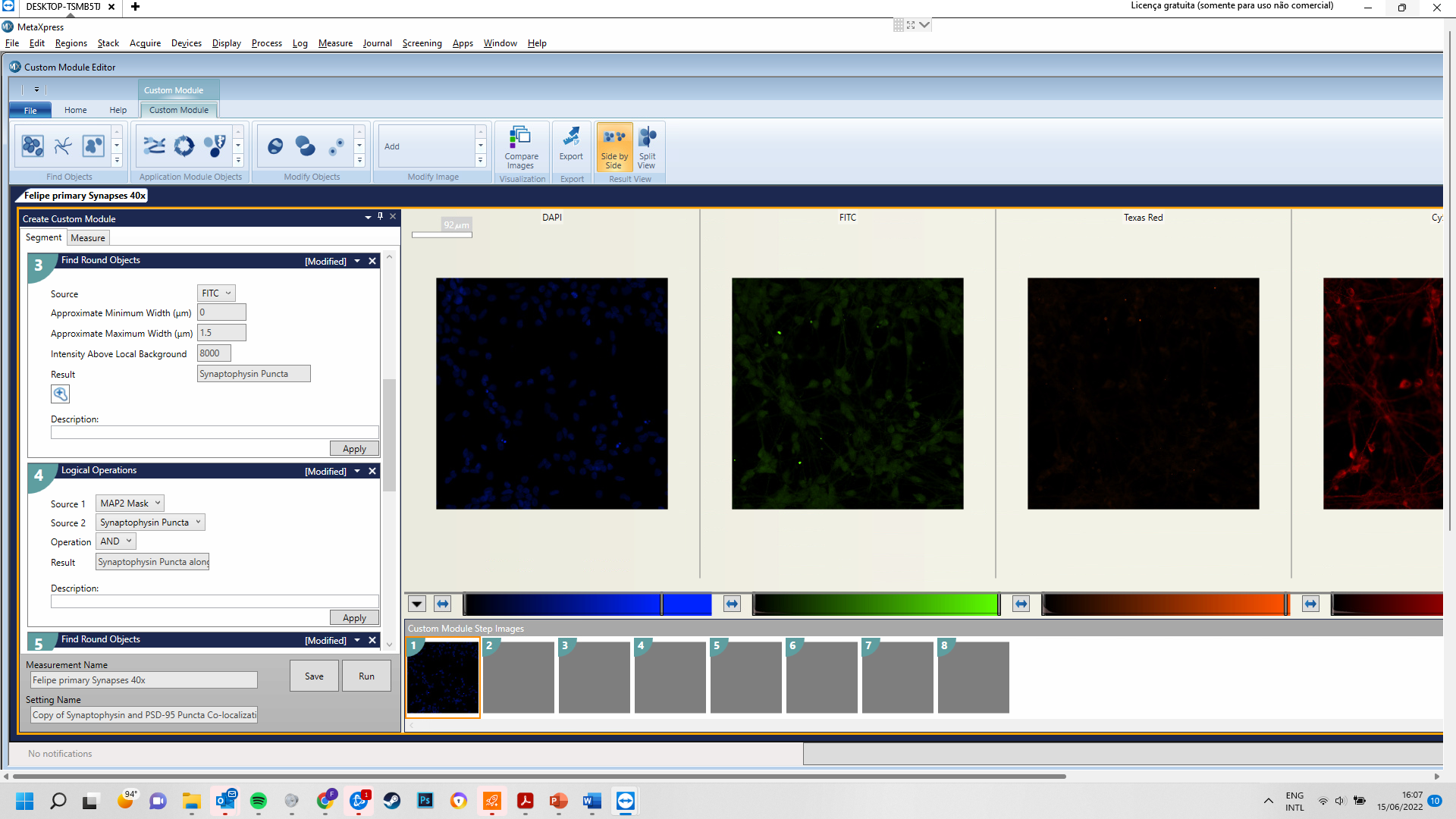

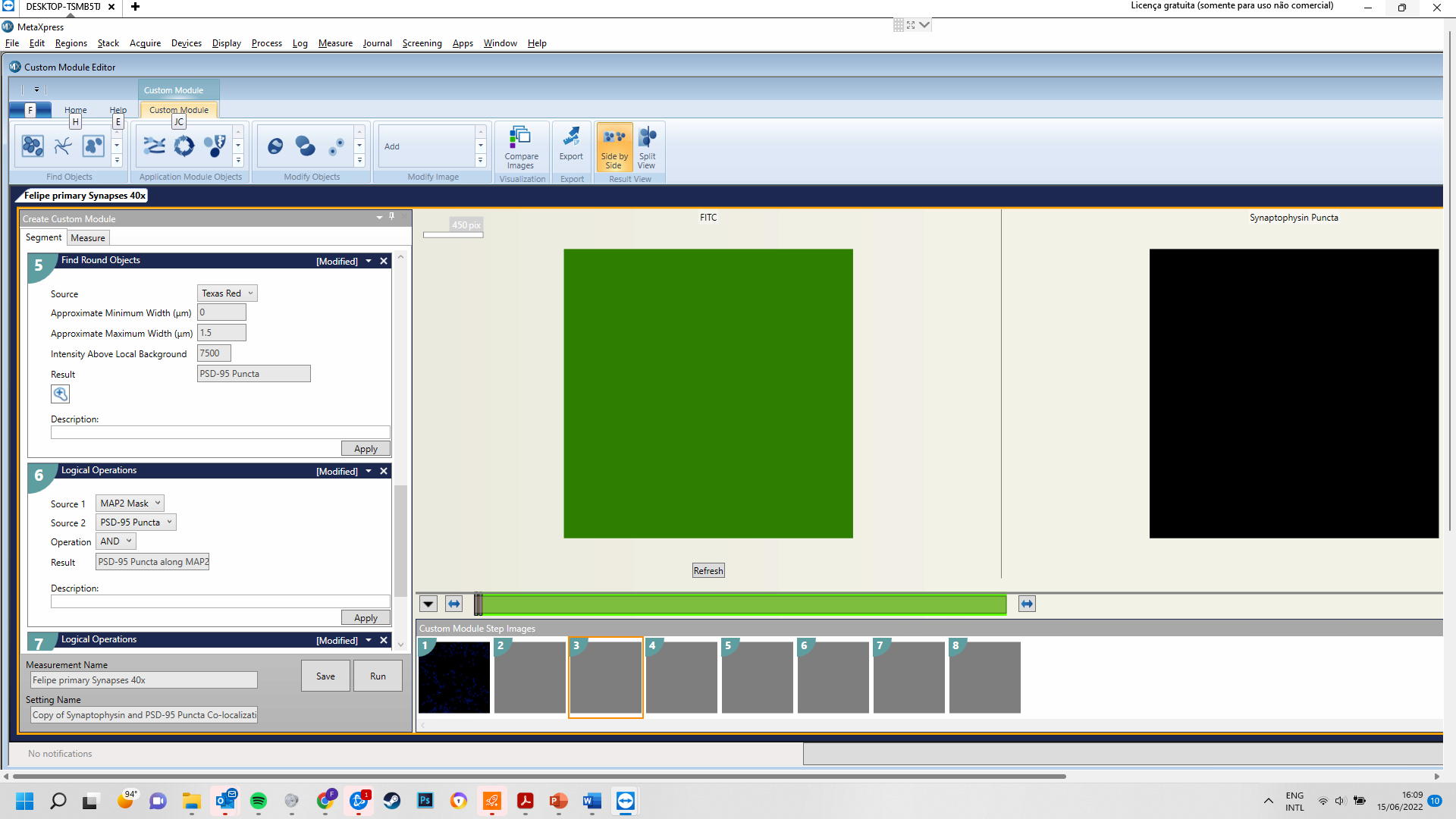

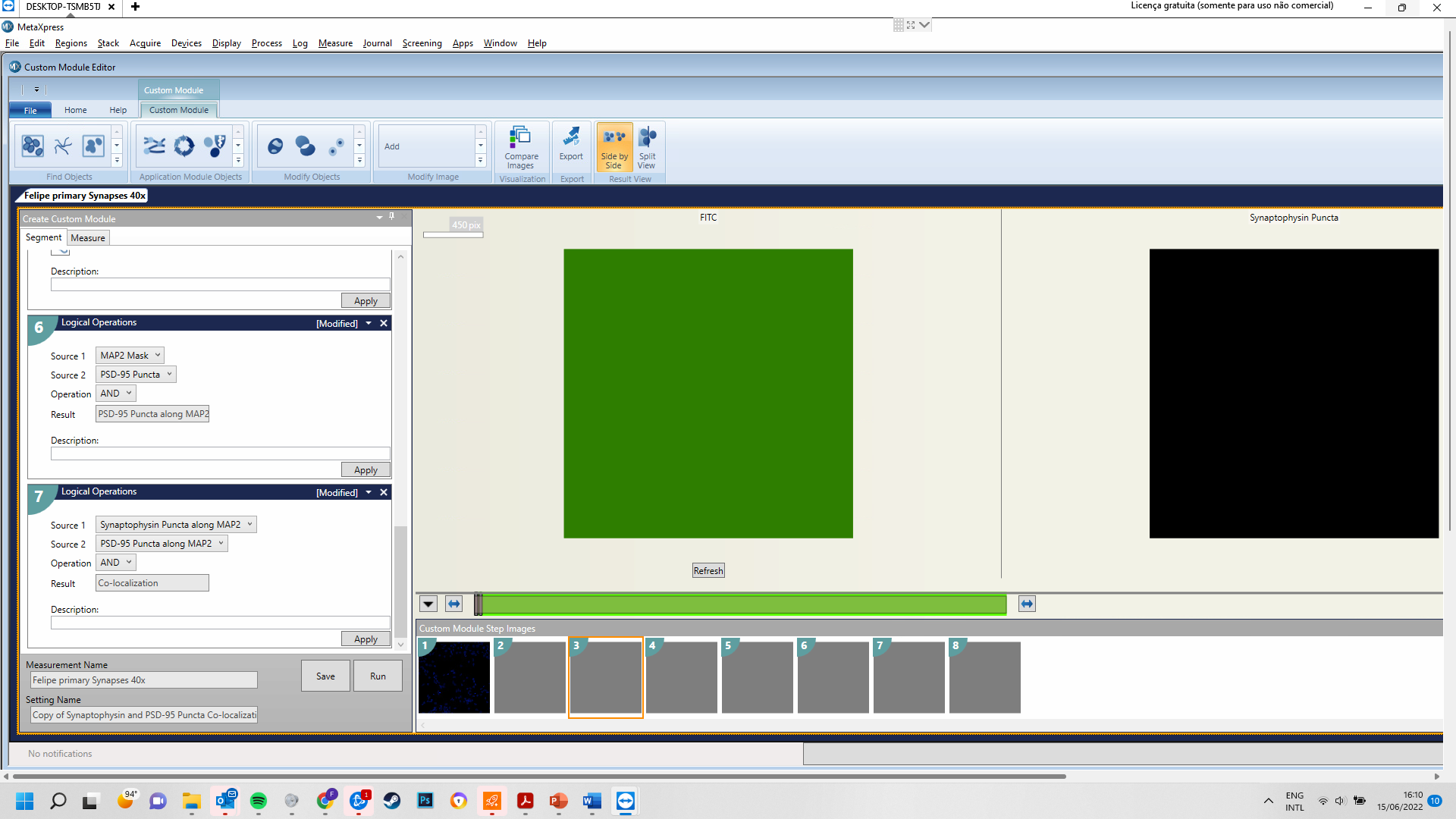

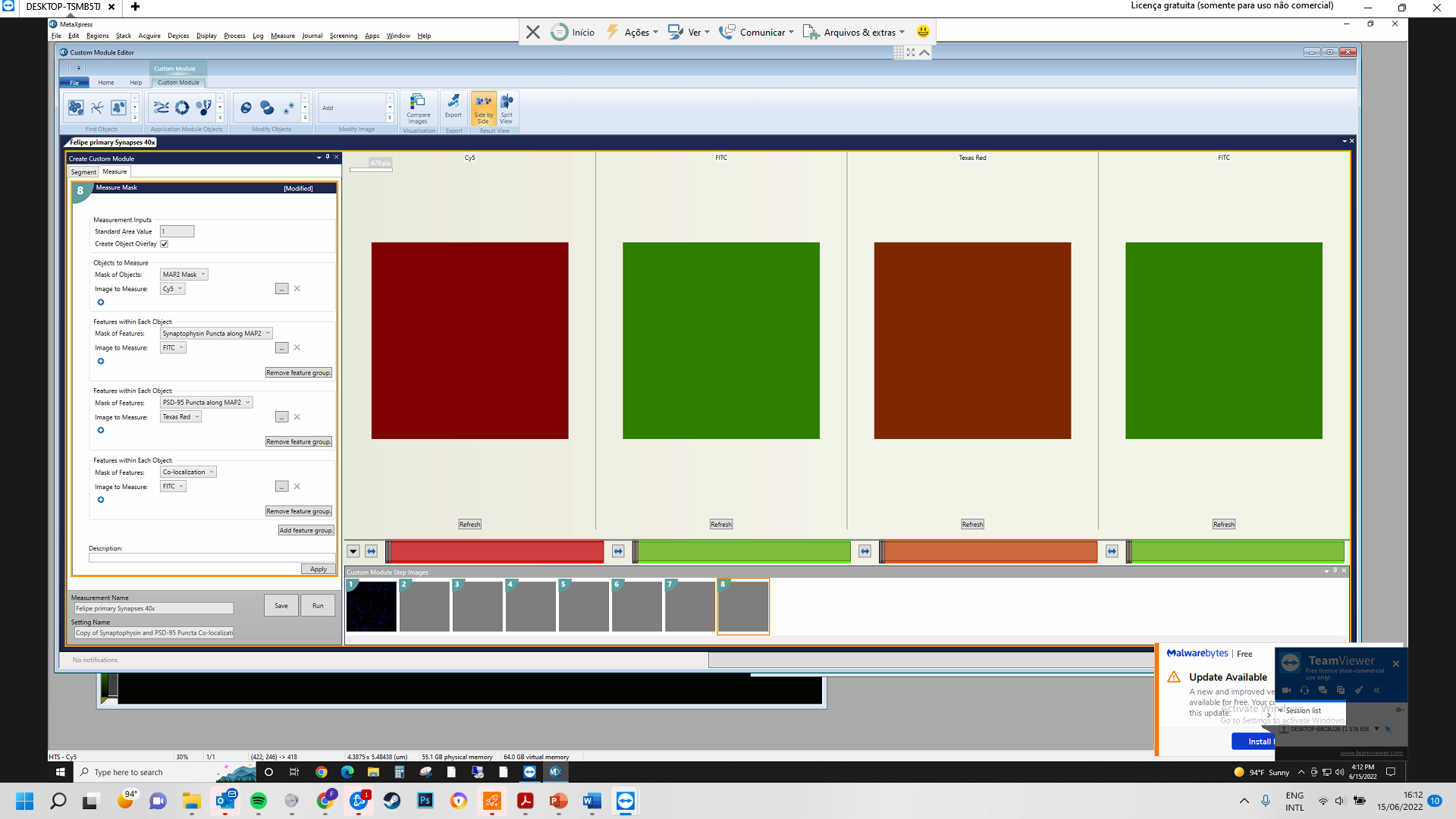

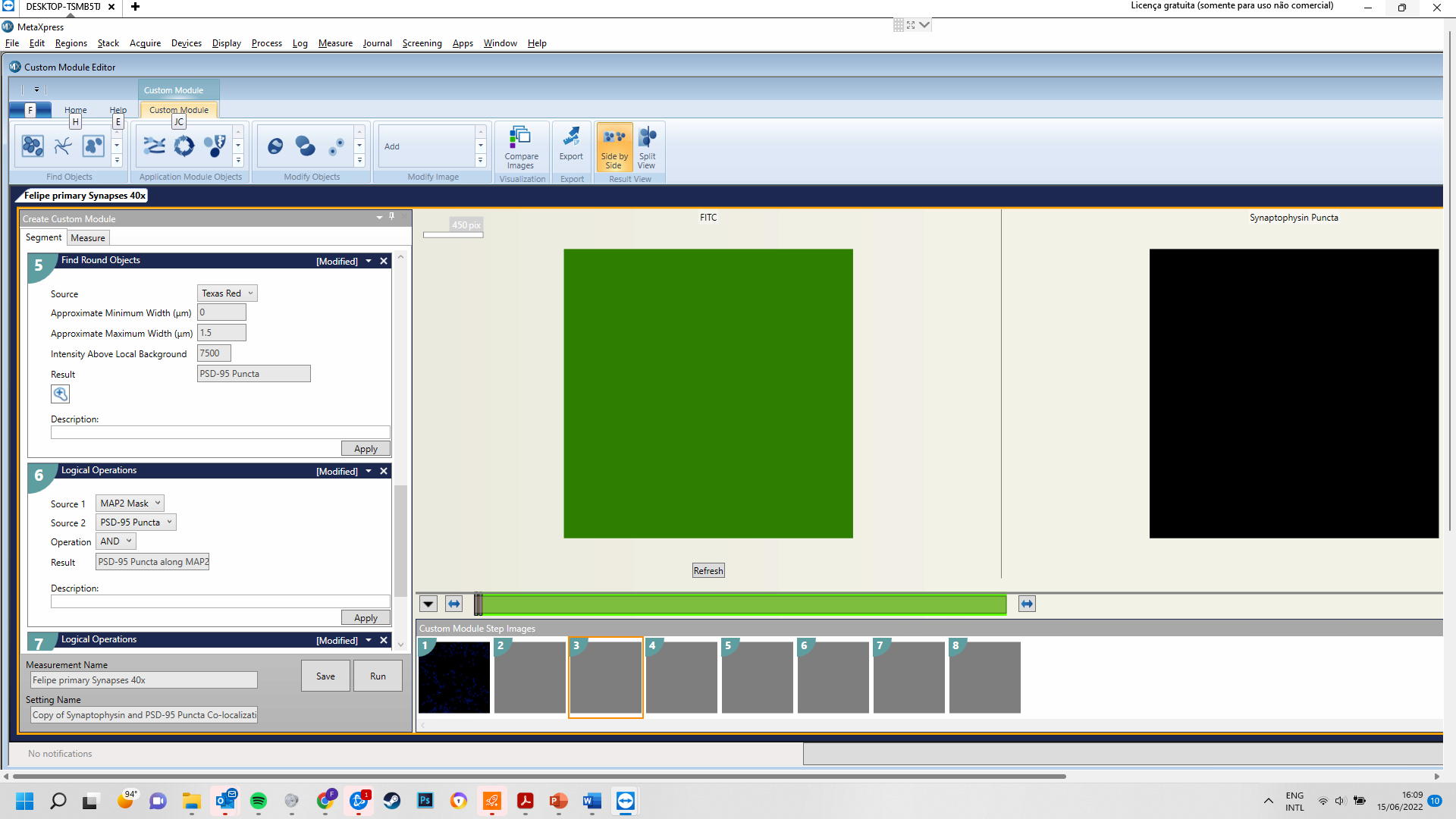


Fig S13. Example of the synaptic connectivity analysis built using MetaXpress Custom Module Editor (version 5.3.0.5).

**References:**

Schmuck, M. R., Keil, K. P., Sethi, S., Morgan, R. K., & Lein, P. J. (2020). Automated high content image analysis of dendritic arborization in primary mouse hippocampal and rat cortical neurons in culture. *Journal of Neuroscience Methods*, *341*(April), 108793. https://doi.org/10.1016/j.jneumeth.2020.108793
